# Matching the Sensory Analysis of Serpa PDO Cheese with the Volatile Profiles—A Preliminary Study

**DOI:** 10.3390/foods14091509

**Published:** 2025-04-25

**Authors:** Antónia Macedo, Maria João Carvalho, Elsa Mecha, Leonor Costa, António Ferreira, Rita S. Inácio, Maria do Rosário Bronze

**Affiliations:** 1 Instituto Politécnico de Beja, Campus do IP Beja, Rua Pedro Soares, 7800-295 Beja, Portugal; atmacedo@ipbeja.pt (A.M.); joaobcarvalho@ipbeja.pt (M.J.C.); rita.inacio@ipbeja.pt (R.S.I.); 2 MED—Instituto Mediterrâneo para a Agricultura, Ambiente e Desenvolvimento da Universidade de Évora, Pólo da Mitra, Apartado 94, 7006-554 Évora, Portugal; 3 CIEQV Centro de Investigação em Qualidade de Vida, Instituto Politécnico de Santarém, Av. Dr. Mário Soares, 110, 2040-413 Rio Maior, Portugal; 4 LEAF (Linking Landscape Environment Agriculture and Food), Instituto Superior de Agronomia, Universidade de Lisboa, Tapada da Ajuda, 1349-017 Lisboa, Portugal; 5 iBET, Instituto de Biologia Experimental e Tecnológica, Av. da República, Apartado 12, 2781-901 Oeiras, Portugal; emecha@ibet.pt (E.M.); leonor.costa@ibet.pt (L.C.); antoniof@ibet.pt (A.F.); 6 Instituto de Tecnologia Química e Biológica António Xavier, Universidade Nova de Lisboa, Av. da República, 2780-157 Oeiras, Portugal; 7 CBQF Centro de Biotecnologia e Química Fina—Laboratório Associado, Escola Superior de Biotecnologia, Universidade Católica Portuguesa, Rua Diogo Botelho, 1327, 4169-005 Porto, Portugal; 8 Faculdade de Farmácia, Universidade de Lisboa, Av. das Forças Armadas, 1649-019 Lisboa, Portugal

**Keywords:** Serpa PDO cheese, volatile composition, sensory analysis, gas chromatography, mass spectrometry, multivariate analysis

## Abstract

Serpa cheese, a Portuguese Protected of Denomination Origin (PDO) cheese, known for its unique sensory attributes, is made from the raw milk of native sheep. In this preliminary work, ten samples of Serpa cheese were submitted for a sensory evaluation performed by an expert panel in a sensory laboratory accredited according to ISO 17025 for Serpa cheese parameters, and the panelists classified the cheeses based on texture, taste and odor scores, in accordance with the specifications for the classification of this type of cheese. All cheeses were analyzed by SPME-GC-MS. Following an exploratory unsupervised multivariate analysis, the supervised multivariate analysis by partial least squares—discriminant analysis (PLS-DA), associated the relative percent area of the identified volatiles with the classification of cheeses attributed by the sensory panel. Among the 144 compounds putatively identified, there was a pattern of compound distribution of some of them, such as acetoin, diacetyl, and 2,3-butanediol, leaning toward the cheese samples with high taste and odor scores, while other compounds, such as ethyl caprate, capric acid, and 3-methylindole, were more associated with the cheese samples rated with a low score. Despite the reduced number of samples that may have imposed some restrictions on the conclusions drawn, there was a clear trend in the volatiles’ distribution, allowing us to identify, based on the higher correlation loadings, potential candidates for the Serpa cheese sensory quality. This preliminary study presents, for the first time, an overview of the volatiles that are present in Serpa PDO cheese that may be responsible for the positive or negative sensory evaluation of this PDO cheese.

## 1. Introduction

Cheese is a food product obtained from milk coagulation in which the whey protein/casein ratio does not exceed that of the milk. It can be obtained by coagulating fully or partially the protein of milk, through the action of rennet or other suitable coagulating agents, and by partially draining the whey produced in the coagulation to increase the final protein content of the cheese [1]. Enzymatic and microbiological reactions take place, transforming the curd into cheese with different ripening stages associated with different volatile profiles. Cheese characteristics depend on different parameters, such as the animal species, the pre-processing method applied to the milk, the coagulation method, the texture, the ripening time, the fat content in the dry product, and the type of microorganisms [2,3]. For artisanal cheeses, like ewe cheeses, the diversity is especially enhanced by the inclusion of strong seasonal variations in milk production, distinct types of animal feed, and human factors [4,5].

The flavor of cheese is determined by the perception of a wide variety of volatile and non-volatile compounds. Several factors related to the processing can affect the final flavor of cheese and its quality, namely load and type of microbiota, pH, redox potential, casein and fat content, micellar size, genetic polymorphism of caseins, calcium content, and thermal treatments [6]. Flavor is an important characteristic that influences consumers’ perception and commercial acceptance [7], as consumers’ preference is driven by the combination of active volatile compounds (AVCs) perceived in the orthonasal system and/or retronasal cavity [8]. Olfactory perception is a complex biological phenomenon triggered by AVCs, usually hydrophobic, which interact with odoriferous receptors in the olfactory epithelium of the nasal cavity [9]. Defects known as off-flavors decrease the cheese quality and lead to financial losses for the producers [10,11,12]. An accurate chemical characterization of AVCs, which can act as potential sensory stimulators, is important to understand which compounds may be considered as key odorants. The cheese volatile compounds, formed during the ripening process, result from the metabolism of residual lactose, lactate and citrate, lipolysis and fatty acid metabolism, proteolysis, and amino acid catabolism, contributing to the presence of carboxylic acids, alcohols, aldehydes, esters, and terpene-based compounds [13]. Metabolic processes are carried out by the starter and non-starter microbiota, enzymes from the coagulant (chymosin, cardosins, and other proteinases), enzymes from the milk itself (plasmin, cathepsin, lipases, and somatic cell enzymes), and by the starter and non-starter lactic acid bacteria [14,15]. The lactic fermentation of residual lactose and the citrate’s metabolism give rise to several cheese flavor compounds, namely lactate, pyruvate, acetate, ethanol, diacetyl, acetoin, 2,3-butanediol, and acetaldehyde [16,17]. Proteolysis, involved in the casein’s hydrolysis, generates peptides and insoluble substances which are subsequently degraded to free amino acids. The catabolism of free amino acids, in turn, produces several compounds, including ammonia, amines, aldehydes, phenols, indole and alcohols, all of which contribute to the flavor of all cheese varieties, but are particularly significant in ripened cheeses [18]. Sulfur-containing amino acids can undergo more extensive degradation, leading to the formation of several AVCs (e.g., methanethiol and other sulfur derivatives). Milk lipolysis, responsible for the release of short- and medium-chain fatty acids, also affects the organoleptic properties of the cheese. The abundance of free fatty acids (FFAs) is directly influenced by the pH. At a high pH, FFAs are less active and often perceived as “soap”, because they are converted into non-volatile salts. At a low pH, FFAs, in high concentrations, are perceived as rancid [17,19]. FFAs are the precursors to methyl ketones, secondary alcohols, aldehydes, lactones, and esters, thus contributing indirectly to the development of flavor [20,21,22]. High levels of FFAs and amines are responsible for the bitter taste of cheese, and some esters, characterized by high thresholds of perception, are associated with sweet and fruity flavors [23,24,25].

Serpa cheese is an artisanal, ripened, regional Portuguese cheese with a Protected Designation of Origin (PDO), typical of the Baixo-Alentejo region in Portugal. This cheese is manufactured in agreement with the *Specifications Book of Serpa Cheese* [26]. It is the result of the coagulation of raw ewe milk produced from the raw milk of native sheep from the region and an aqueous extract of dry thistle flowers (*Cynara cardunculus* L.) as a coagulant [27]. Acid proteinases, called cardosines, the enzymes of greatest importance present in the flowers of *C. cardunculus* L., have a strong proteolytic action which leads to the extensive decomposition of caseins, producing cheeses characterized by a soft, buttery texture with a typically spicy taste [28]. Cheeses produced from raw milk have a more intense and characteristic flavor, because when the milk is heat-treated, there is a reduction in microorganisms and inactivation of enzymes, leading to a lower production of volatile compounds [6,29,30].

The production of Serpa cheese is of economic interest for the development of the region, and its quality is regularly monitored for cheese valorization. So far, existing studies underscore the relevance of volatile organic compounds in understanding the sensory attributes and quality of Serpa cheese, focusing mostly on broader aspects of cheese production, preservation [31], and microbial interaction [32]. This preliminary study intends to evaluate the volatile composition of Serpa PDO artisanal cheeses, establishing an association with sensory characteristics evaluated by a expert sensory panel in an accredited laboratory. Following an untargeted metabolomic approach, a complete database of putatively identified volatile compounds potentially associated with the quality of the cheese was created. Although a small number of samples were analyzed, this preliminary approach will contribute valuable insights into the desirable taste and odor profiles of Serpa PDO cheese, such insights informing production improvements, the control of the ripening process, and/or the preservation of traditional and unique attributes. In future work, more samples must be considered to confirm the results presented here.

## 2. Materials and Methods

### 2.1. Samples

The samples of Serpa PDO cheese (n = 10) were provided by the certification body (CERTIS—Control and Certification, Lda). The samples were collected from local producers (origin not identified for impartial evaluation) that comply with the specifications related to the manufacturing process described in the specification rules for Serpa PDO cheese and transported to the laboratory on the same day, under temperature-controlled conditions (0–10 °C). The samples were received, inspected for possible anomalies, coded, and stored in the fridge at a controlled temperature of 3–5 °C for 2 days. Subsequently, samples were analyzed by the sensory panel and then subjected to volatile compound analysis.

### 2.2. Methods

#### 2.2.1. Sensory Analysis Evaluation

Sensory evaluation of the samples was conducted in the Sensory Analysis Laboratory of ESA/IPBEJA. The Serpa cheeses (n = 10) were evaluated by twelve assessors (ten females, two males, aged 28–60 years) who were selected, trained, and monitored according to international standards [33]. All sessions were conducted at room temperature (20–22 °C) in a sensory room free from external smells, noises, and distractions, and equipped with eight booths with technical characteristics adequate for sensory analysis, including a white 6500 K light and a sink, according to international standards [34]. The laboratory is accredited, according to ISO 17025, for the sensory evaluation of Serpa cheese [35], being annually audit by Portuguese Institute for Accreditation (IPAC) with Accreditation Annex nr.L0685-1.

Prior to sensory evaluation, entire cheeses were kept for 2 h at room temperature (20 to 22 °C). For the visual aspect attribute evaluation, half of the cheese was presented to all the assessors. Cheeses were cut into representative triangular slices (15 to 20 g) without removing the rind, presented in a Petri plate, coded with a random three-digit code, and evaluated in a randomized order in the booths. The assessors were instructed to clean the palate after each sample evaluation with a cracker and spring water. The following sensory attributes were analyzed: crust, shape and consistence, texture and paste color, and, finally, taste and smell, according to Table 1, presented in a scoresheet. The scale used for classification was divided into a minimum interval of 0.5. Regarding the crust, for example, the scores were as follows: 0.0, 0.5, 1.0, 1.5, 2.0, 2.5, 3.0, 3.5, and 4.0, as indicated in Table 1. It is important to note that the parameters “texture and paste color” and “taste and smellr” were more valorized in the evaluation, with a maximum score of 6.0. As described previously, the first three parameters were evaluated by analyzing half of the cheese on a side bench of the sensory laboratory, individually and without crossing paths with another assessor. The last parameter, taste and smell, was evaluated in an individual booth.

According to the book of specifications, it is required for a sample to obtain a minimum score of 4 (average of the assessors’ scores) with respect to the taste and smell parameter and a score of 14 as the sum of the four parameters for the cheese to be certified as PDO.

#### 2.2.2. Volatile Composition Analysis

##### Sample Preparation

A portion of sample (6.5 g) was removed from the inside of the cheese (±1.5 cm from the surface), introduced into a 20 mL glass vial, and covered with silicone PTFE red 16 mm septum (BGB Analytik, Specanalítica, Portugal). Each sample was analyzed in triplicate.

##### Analysis by SPME-GC-MS

Solid-phase micro extraction (SPME) was used as the sample preparation methodology for the analysis of the volatile compounds. A divinylbenzene/carboxen/polydimethylsiloxane (DVB/CAR/PDMS) fiber from Supelco (Bellefonte, CA, USA), 23 Ga, 50/30 μm, was used. Samples were heated for 40 min at 40 °C, with an agitation speed of 250 rpm, and injected at 250 °C. The time of desorption from the fiber was 5 min, and the sample injection was conducted in a splitless mode. An AOC-5000 Shimadzu auto-sampler (Shimadzu, Tokyo, Japan) was used. GC–MS analysis was conducted on a QP 2010 Plus, Shimadzu, Kyoto, Japan, equipped with a TeknoKroma Sapiens Wax-MS column, 60 m × 0.25 mm i.d., film thickness of 0.25 μm. A gradient of temperature was used for the separation of a sample’s components: the column temperature was initially maintained at 40 °C for 5 min, then increased, at a rate of 5 °C per minute, to 170 °C, a temperature that was then increased to 230 °C at a rate of 30 °C per minute, and maintained at 230 °C for 4 min. Then, the temperature was programmed to increase to 260 °C at a rate of 30 °C per minute, and this temperature was maintained for 2 min. The total time for each analysis was set to 40 min. Helium was used as the carrier gas, with a flow rate of 4 mL/min. For MS conditions, a mass range of 29–300 *m/z* was used. The electron ionization (EI) energy was 70 eV, the ion-source temperature was set to 245 °C, and the interface temperature to 250 °C.

##### Identification of Compounds by GC-MS

The Shimadzu software, GCMS solution, version 4.50 SP1, was used for data acquisition and analysis. The putative identification of the compounds was assigned by comparison of their linear retention index (LRI) with values in the literature, and GC–MS spectra were compared with spectra from the libraries NIST and Wiley [36]. The determination of LRI was achieved using a mixture of alkanes, C8 to C20, commercialized by Merck (the retention times are shown in Table S1 in Supplementary Files), in accordance with Equation (1) [37]:

(1) $$LRI=100\left(n+\left(T_{r}\left(x\right)-T_{\left(r\left(n\right)\right)}\right)/\left(T_{\left(r\left(N\right)\right)}-T_{\left(r\left(n\right)\right)}\right)\right)$$

where *LRI* = linear retention index *x*; *T_r_*(*x*) = retention time of the compound *x*; *n* = number of atoms of the nearest smaller alkane; *T*_*r*(*n*)_ = retention time of the smallest alkane; *T*_*r*(*N*)_ = retention time of the nearest large alkane.

#### 2.2.3. Data Analysis

The mean scores attributed by the assessors to the different cheese samples were subjected to one-way ANOVA and cluster analysis, using SPSS v. 10.0 (SPSS Inc., Chicago, IL 60611, USA) to check for any assessors with outlier scores.

For panel internal quality control, blind samples were evaluated per session. A means comparison test (*Scheffé*) was performed to evaluate the members’ performance. If each member did not significantly discriminate (*p* > 0.05) between cheeses used as blind samples, the performance was considered to be reliable within the panel and to have good repeatability. These procedures are mandatory for an accredited panel, according to ISO 17025.

To evaluate significant differences among the families of compounds analyzed and detected in the cheeses, a one-way ANOVA with a post-hoc Scheffe’s test was performed. A multivariate exploratory analysis of the data was conducted by principal component analysis (PCA) based on the correlation data matrix. With this analysis, the samples were distributed in a biplot based on the established correlations between the variables, using Pearson’s R correlations. This analysis retains the data information while reducing their dimensionality to two components, showing trends and patterns in the samples’ distribution. The PCA analysis was performed in SPSS v. 10.0 (SPSS Inc., Chicago, IL 60611, USA). Following the PCA unsupervised data analysis, a supervised approach was adopted by using partial least squares—discriminant analysis (PLS-DA), which helped emphasize the differences among the samples and understand possible correlations between the volatile compound percent area (predictors) and the groups established based on the panel mean scores for taste and smell sensory analysis (response variable). For this analysis, the *Unscrumbler* software (version 10.4.1), AspenTech (Bedford, MA, USA), was used, and the kernel algorithm was applied to the PLS analysis. Predictor variables with correlation loading values higher than 0.5 were considered potential contributors to sensory appreciation of Serpa cheese quality.

## 3. Results and Discussion

### 3.1. Serpa Cheese Classification Based on Sensory Analysis

As mentioned previously, to be accepted as a Serpa PDO certified cheese, the sensory evaluation requires a minimum of 4.00 in the taste and smell attribute and a final classification (final score) of a minimum of 14.00 [26]. Cheeses with a classification of taste and smell equal to or higher than 4 may be evaluated as high-score cheeses and considered cheeses that can use the denomination. The others, with a classification lower than 4, are classified as cheeses of lower quality that cannot carry the denomination. These criteria are defined in the specification book and must be followed for the evaluation of the cheeses and its certification [26].

The quality of Serpa cheese samples (n = 10) evaluated by the sensory panel is presented in Table 2 and was defined based on the assessors’ taste and smell appreciation.

Despite some of the cheeses being evaluated with a final score higher than 14.00, as presented in Table 2, they were classified as low-score cheeses because their taste and smell values were lower than 4.00 (between 3.60 and 3.86). The other cheeses, with a final score ranging from 14.70 to 17.45 and a taste and smell quality between 4.25 and 5.25, were classified as high-score cheeses by the sensory panel.

### 3.2. Volatile Composition by SPME-GC-MS

**Figure 1:**
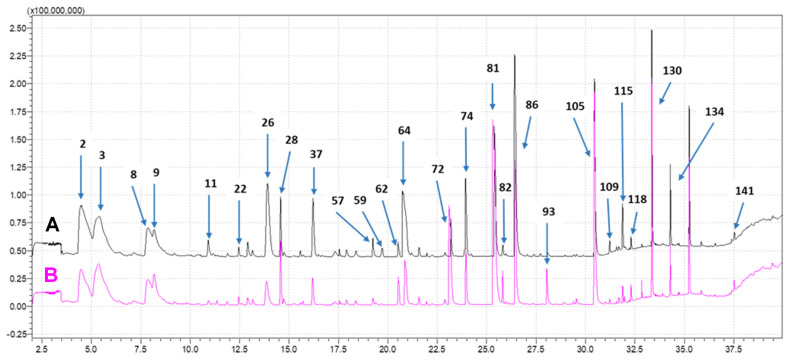
Example of chromatographic profiles obtained through SPME-GC-MS for the Serpa cheese samples, (**A**) cheese 110 (black) and (**B**) cheese 64 (pink), classified, respectively, as having the highest and the lowest sensory quality scores. The numbers highlighted in the figure indicate the compounds tentatively identified in Table 3.

The solid-phase microextraction—gas chromatography—mass spectrometry (SPME-GC-MS) methodology may be used to characterize the volatile composition of different types of samples. In this sample preparation methodology, volatile compounds are adsorbed or absorbed, depending on the type of fiber used, onto the surface of the fiber in contact with the headspace generated inside the bottle where the sample is kept. After analysis by GC-MS, the tentative identification of the compounds, responsible for the different flavors in the cheese samples, was achieved by comparison with online mass spectral libraries for volatile compounds from the National Institute of Standards and Technology (NIST) and Wiley [38]. The SPME-GC-MS analysis of the Serpa cheese samples (Table 3) allowed for the tentative identification of 144 compounds. Figure 1 shows an example of a comparison between the chromatographic profiles of the cheese sample that obtained the highest score from the sensory panel (sample 110) and the sample exhibiting the lowest score (sample 64). The compounds highlighted with numbers were sorted in ascending order of retention time, in accordance with Table S1 (in the Supplementary Files). Table S2 presents the experimental linear retention indexes and their comparison with the values defined in the literature for all the identified compounds.

In Table 3, the tentatively identified compounds are shown and organized into different families. For each compound, the relative area was calculated as (peak area/total chromatogram area) × 100.

The volatile compounds presented in Table 3 belong to different chemical families, such as ester, carboxylic acids, alcohols, cyclic hydrocarbons, ketones, and furans, with 37, 25, 21, 16, 15, and 10 different volatiles present in each family, respectively, as detailed in the Supplementary Files (Table S2).

Table 3 also presents the sum of the average areas for the different families of volatile compounds. Although producers must follow the book of specifications, the cheeses are produced in an artisanal way by small- and medium-sized enterprises, dispersed across the Baixo Alentejo region, within the demarcated region of PDO Serpa cheese. Therefore, variations in cheese quality could be attributed to differences in the cheesemaking process, including the milk (within raw ewe milk from geographic zone at different lactation stage, animal age, number of parities) and the endogenous microbial communities (raw milk is used without thermal treatment). The weather conditions, with an uneven distribution of annual rainfall, characterized by abundant water in autumn and winter and significant dryness during summer, may affect forage production, requiring animals’ diet supplementation with feed, which ultimately affects ewe’s milk production and quality.

In addition, given the artisanal manufacturing process, inadequate ripening may occur if ripening chambers, with controlled temperature, relative humidity, and air speed conditions, are not well regulated. Under these conditions, anomalous biochemical processes may arise, producing cheeses that do not meet conformity standards [39]. The comparison analysis showed that, despite the similarity in the distribution pattern, in high-score cheeses, the ketone family showed a higher relative area, mostly due to the greater abundance of compounds such as 2-butanone (#2), diacetyl (#4), butyl acetone (#22), and acetoin (#37). In low-score cheeses, some specific terpenes like eucalyptol (#24), D-limonene (#23), esters, ethyl caprate (#82), propyl decanoate (#91), and some carboxylic acids such as n-capric acid (#134), caproleic acid (#135), lauric acid (#41), and myristic acid (#44) were predominant, despite their lower percent relative area. In addition, the worst-rated cheese (64) presented a higher relative area for carboxylic acids, mainly due to propanoic acid (#72), butanoic acid (#81), caproic acid (#105), and valeric acid (#93). The higher percentage of some carboxylic acids was previously described in samples of putrid Turkish cheese [40]. Among these, propanoic acid (#72) was present in a significant amount, and it is responsible for adding fecal notes to the flavor profile [11,41]. Butanoic acid causes a cheesy and sharp aroma and can originate from lipolysis by endogenous milk or microbial-origin lipases, or even result from the metabolism of deamination of amino acids and lipid oxidation [42,43]. The presence of excessive acids can be attributed to the excessive activity of lactic acid bacteria, yeasts, and natural microbiota from the raw milk [44].

Terpenes, furans, and lactones are known to be responsible for a characteristic cheesy flavor. The terpenes may derive from the sheep’s diet, as described elsewhere [45], and the lactones, which are cyclic compounds formed via the intramolecular esterification of hydroxy fatty acids, are known to contribute to the intense flavor of long-matured cheese [46]. Concerning the results for lactones, their contribution in terms of peak area was low and not discriminative.

Regarding the residual volatile compounds, whose total area was lower than 1%, the trisulfide family, composed of dimethyl trisulfide (#55), was only detected in some of the samples classified as having a low score (cheese samples 64 and 125). The reduced number of samples contributed to this variation in volatile profiles. The sulfur compounds, usually formed as a result of sulfur amino acid catabolism, are associated with flavor defects, as described elsewhere [46], or very ripe cheeses, with odor notes of cooked cabbage, broccoli, or cauliflower [47]. Nevertheless, in the Serpa cheese samples with higher taste and smell scores (cheese samples 110 and 679), there was a higher relative area of the sulfone, namely dimethyl sulfone, indicating that specific sulfur derivative compounds may play an important role in the Serpa cheese flavor. Regarding the sensory evaluation performed by the assessors, it is important to stress that, due to different odor thresholds, regardless of the relative area of the volatiles, the volatiles’ sensory impact can be perceived and integrated differently, introducing variability in the sensory evaluation. To identify the existence of trends in the volatile composition of the two perceived classes of Serpa cheese, a supervised PLS-DA analysis was conducted. This statistical tool allowed for the identification of compounds with a higher contribution to the discrimination of the two sample groups, regardless of being key odorants or not.

### 3.3. Correlation Between Serpa Cheese Volatile Composition and the Sensory Quality Evaluation

**Figure 2:**
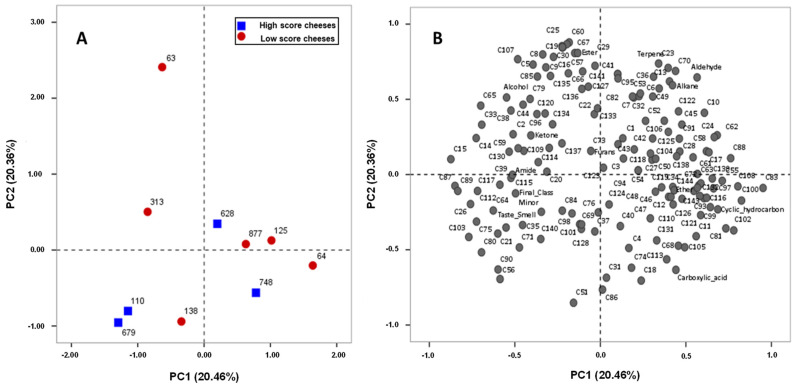
PCA analysis showing the distribution of the cheese samples (110, 679, 748, 628, 125, 63, 313, 877, 138, and 64) in a space defined by the two principal components, which together explain 41% of the total variance (**A**). For this analysis, the quantitative variables included the relative area of the individual volatile compounds, the relative area of the compounds’ families, and the sensory evaluation scores. (**B**) Projection of the considered variables in the loading score plot. The cheese groups defined by the assessors were superimposed on the (**A**) plot.

The exploratory PCA analysis, based on the correlation data matrix (Figure 2 and Table S3), demonstrates the correlation between the relative area of the different volatiles and the sensory quality evaluation quantified as taste and smell scores and final classification scores by the assessors. As shown in Figure 2A, due to the reduced number of samples in the analysis, no clear distribution pattern was defined in the 2D space.

**Figure 3:**
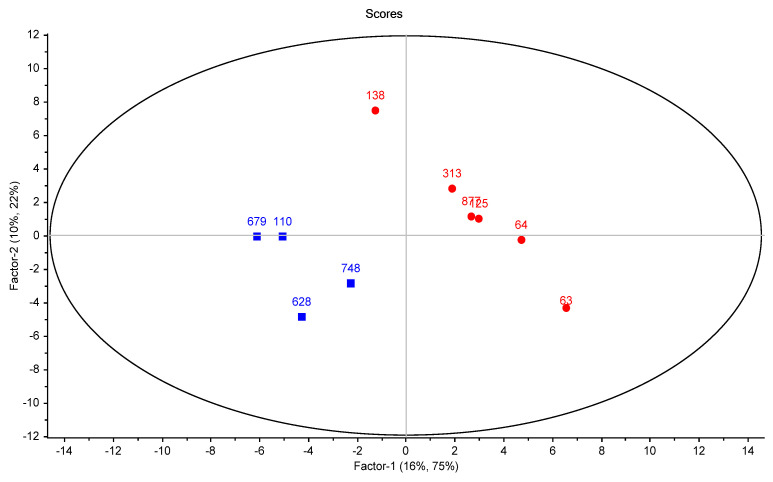
PLS-DA score plot showing the distribution of the cheese samples (110, 679, 748, 628, 125, 63, 313, 877, 138, and 64) in a space defined by the two first factors. For this model, the relative area of the volatile compounds (average value for the analytical triplicates) was used as the predictor variable (accumulated explained variance of 26%) and the groups established based on the sensory analysis scores (high score—H ■, higher than 4; low score—L ●, lower than 4) were used as the response variable (accumulated explained variance of 97%).

Although no distribution pattern was determined by the PCA, the ability of the PLS-DA analysis to identify sharp differences among samples was visible in the conducted supervised PLS-DA analysis (Figure 3), which clearly separated the cheese sample groups, defined based on the evaluation performed by the sensory panel: samples with a score higher than 4 (H) and samples with a score lower than 4 (L). The two established groups of cheese samples were separated along factor 1, which explained 75% of the variance.

**Figure 4:**
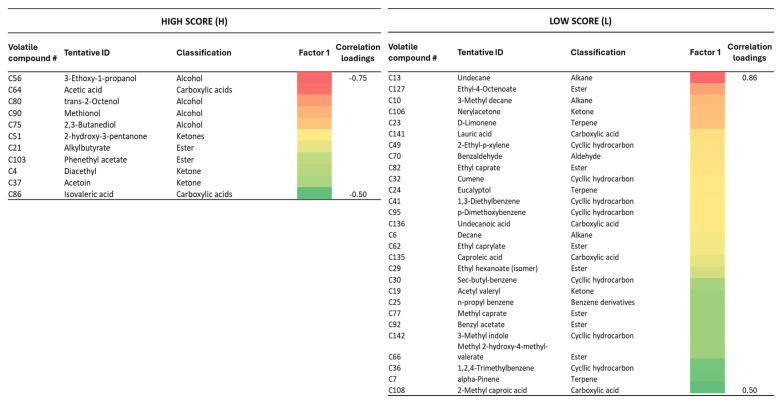
Heatmap representation of the correlation loadings in factor 1. Only volatile compounds with correlation loading values higher than |0.5| are represented. For the group of samples classified as having a high score, the correlation loadings were sorted in ascending order (0.747 to −0.501), while for the low-score samples in descending order (0.857 to 0.509). Visualization of the correlation loadings absolute values, considering that warmer tones like red indicate higher values, while cooler tones like green represent lower values.

Based on the correlation loadings obtained from the PLS-DA analysis for factors 1 and 2 (Table S4), the two groups of Serpa cheese samples classified with high (H) and low (L) sensory quality scores were discriminated mostly by the relative area of the volatile compounds, highlighted in Figure 4.

These data suggest that some volatile compounds with correlation loadings higher than |0.5| can have a higher discriminatory ability and may represent potential candidates for the distinct Serpa cheese sensory quality evaluation.

Diacetyl (#4) and acetoin (#37) are aromatic compounds associated with higher taste and odor scores, and represent major volatile compounds formed by the action of *Lactacoccus lactis* via aspartate and alanine catabolism. Both are responsible for the creamy and buttery notes of the cheese [48], in accordance with the expected, moderate buttery texture of Serpa cheese [26]. As shown in Table S3, compounds such as phenylacetonitrile (#117), 2,3-butanediol (#75), and alkyl butyrate (#21) showed a high positive correlation (>0.7) with desirable flavors, indicated by higher classification scores in the cheese sensory evaluation. These compounds are produced during the fermentation process by different microorganisms, yeasts, and lactic bacteria, improving the overall sensorial quality of the cheese. The compound 2,3-butanediol (#75) can be produced by a redox reaction from acetoin (#37) [48].

Isovaleric acid (#86), a carboxylic acid, is obtained from the leucine metabolism [49], and has been described as being responsible for the cheesy flavor characteristic of cheeses manufactured from the milk of small ruminants [49]. This compound has been previously reported as a free fatty acid with a high odor activity value in the Serra da Estrela cheese, and it is known to confer a rancid, cheese-like, and sweet flavoring to sheep cheeses [49]. Other studies conducted on Divle Tulum cheese have also found an increase in the amount of isovaleric acid during ripening [50,51]. The content of that compound, over the storage period, slightly increased in the Tulum cheese samples, ranging from 0.30 to 1.59 mg kg^−1^ [51].

Methionol (#90) is a sulfur-containing volatile compound associated with the cheese flavor that is produced through the transamination of methionine, followed by decarboxylation and reduction reactions [52], and it may contribute to the Serpa cheese appreciation.

Acetic acid (#64) is associated with a vinegary, tangy, and sour taste and has been previously described in the Ezine Turkish cheese [53] as one of the volatile compounds responsible for the flavor profile of many cheeses.

The 2-phenylethyl acetate (#103) is associated with a floral flavor in dairy products and has been described as a potent odorant in rosy/floral Cheddar cheeses [54].

All these different compounds contribute to creating the distinctive sensorial attributes of Serpa cheese, making it a high-quality and flavorful product. Despite their presence in other types of cheese, their concentration and the presence of additional compounds can vary, determining the overall quality and characteristics of the cheese. Concerning the samples with lower taste and flavor scores, there was a high correlation (Pearson’s coefficient correlation < −0.7) with compounds, such as nonanal (#58) and myristic acid (#144), produced as a result of triglyceride breakdown (Table S3). The presence of phenyl ethylacetate (#99), which is responsible for sweet notes, does not align well with the acidic and savory flavor profile desired by the assessors (Pearson’s coefficient correlation <−0.7, Table S3). This compound can be produced during the catabolism of aromatic amino acids by lactic acid bacteria, modifying the balanced flavor of the cheese. Its presence is linked to fermentation problems or cheese spoilage. The presence of terpenes, such as D-limonene (#23), eucalyptol (#24), and α-pinene (#7), derived mostly from the pasture used for feeding, is considered a biomarker of sheep milk authenticity [55], although their higher relative area in the chromatograms of cheeses 63 and 125 was associated with a defect in smell and taste (Table 3).

The esters, such as ethyl-4-octenoate (#127), ethyl caprate (#82), ethyl caprylate (#62), ethyl caproate (#29), methyl caprate (#77), benzylacetate (#92), and methyl 2-hydroxy-4-methylvalerate (#66), seem to contribute to an undesirable flavor in Serpa cheese. In fact, higher concentrations may indicate imbalanced fermentation, overactive esterification, or improper microbial development during cheese aging. Compounds such as ethyl hexanoate and ethyl caprate were related to defective Zamorano cheese [28]. In fact, although esters are responsible for fruity flavors in dairy products, such flavors appreciated in many cheeses like Parmesan or Parrano, they are undesirable in other cheeses. In Cheddar cheese, the presence of ethyl caproate (#29) and ethyl caprylate (#62) has been described as conferring positive odor notes (sweet, pleasant, and brandy notes) [56]. It is accepted that esters are produced by esterification reactions (between free fatty acids and alcohols) catalyzed by esterases/lipases from cheese microbiota. The ester synthesis is reversible and depends on environmental conditions, such as water activity [14,46]. The abundance of esters is related to the ripening status of the cheese [57].

The alkyl benzene compounds detected in the low-score cheeses, namely 1,3-diethylbenzene (#41), sec-butylbenzene (#30), propylbenzene (#25), and 1,2,4-trimethylbenzene (#36), have been reported in other cheeses as degradation products of carotene in milk [58].

Lauric acid (#141) has been reported to be responsible for fatty odors [59,60]. An increase in the presence of volatile free fatty acids (FFAs) is observed during cheese ripening [61] and can be related to progressive lipolysis during maturation [62].

The 3-methylindole (#142) compound, which has negative effects on flavor, results from the catabolism of free amino acids, namely from tryptophan degradation that is produced by bacterial activity, particularly from *Clostridium*, *Bacillus*, and *Enterobacteriaceae* [18,59].

Other compounds, such as 4-methylphenol (#125), are normally formed as a result of an atypical Strecker degradation [10] and present a correlation loading of 0.4856 in factor 1, showing a higher relative area in the lower-scoring cheeses. This result is in accordance with the literature, which indicates a variation in sensory appreciation (sharp, medicinal, and sweet to smoky, plastic, and unpleasant sheep-yard notes), as its concentration increases [15]. It is worth mentioning that some of the volatile compounds, such as ethyl esters (e.g., phenethylacetate), aldehydes (e.g., benzaldehyde), ketones (e.g., diacetyl), amino acids (e.g., glutamic acid), and organic acids (e.g., acetic acid), contributed to the cheese’s taste, with sweet and fruity notes (esters), green and grassy to nutty and sweet notes (aldehydes), buttery and creamy notes (aldehydes), umami taste (amino acids), and tangy notes (organic acids) [63].

Pinpointing the specific compounds causing off-flavors allows for the adjustment of the cheesemaking process by eliminating the sources of unpleasant flavors, ultimately ensuring the safety of the cheese, the high quality of the product, and consumer satisfaction.

## 4. Conclusions

In this preliminary work, although no apparent pattern of separation could be established by PCA among the Serpa cheese samples sensorially classified as different, the use of PLS-DA sharpened the differences among the evaluated Serpa PDO cheeses, contributing, for the first time, to the association of specific volatile compounds with the olfactory appreciation as assessed by an accredited sensory panel. This preliminary selection of tentatively identified compounds, considered to be potential contributors to the sensory quality of Serpa cheese, includes compounds such as acetoin, diacetyl, and 2,3-butanediol. Conversely, compounds such as 3-methylindole, alkyl benzenes, like propylbenzene, terpenes, like eucalyptol, and esters, like ethyl caprate, seemed to be responsible for a lower score in the sensory evaluation of Serpa cheese. Future work should rely on an extended database with a higher number of samples for the development of a robust PLS-DA model capable of predicting/ or tracing back cheese sensory quality. To improve the quality of the cheese volatile compound databases, future studies should be designed to include the physicochemical and microbiological composition of the cheese as well as the preparation conditions, such as the temperature and humidity during storage, and the maturity status of the cheese. The microbiological composition can be studied in the laboratory, but the other conditions that may contribute to the differences in the sensory evaluation of these Serpa PDO cheeses must be controlled for by producers using artisanal processes.

## Figures and Tables

**Table 1:** Sensory attributes, description, and scores used in the evaluation of Serpa PDO cheese scoresheets [26].

Sensory Attribute	Description	Scores
Crust	Flat or wavy rind, thin or medium-thin; whole/continuous rind: with an intense straw-yellow or lemon-yellow and sometimes with dried molds spots.	3.5–4.0
	Little adherent rind, malformed, with difficult cheese paste containment, with slits more or less extended and open, or hard and thick, with a white color, stained, or yellow-brownish intense color.	2.0–3.0
	Deeply deteriorated, excessive thickness and deeply stained.	0.0–1.5
Shape and consistency	Regular with a side bulge and no sharp edges. Semi-soft consistency with some fluctuation—massive sound or slightly tympanic in sound.	3.5–4.0
	Edges with hard consistency, or excessively deformable due to excessive softness; sharp tympanic sound.	2.0–3.0
	Exaggerated deformation; too much fluid consistency.	0.0–1.5
Texture and paste color	Well-bonded, closed, or with some eyes, and a medium buttery paste; white-ivory, uniform color.	5.5–6.0
	Badly bonded, hard center, haggard, or irregular, with interstitial water; white-matte color, white center with irregular coloration and stains.	3.0–5.0
	Not bonded, spongy; color white or stained with different tones.	0.0–2.5
Taste and smell	Smooth taste or slightly sharp and spicy; smooth smell or slightly strong and ammoniacal.	5.5–6.0
	Soapy, salty, bitter, strong, and unpleasant, strong and sharp ammoniacal smell.	3.0–5.0
	Disgusting taste and smell.	0.0–2.5

**Table 2:** Cheese sample classification based on the sensory analysis evaluation.

Serpa Cheese ID	Classification (Final Score)	Classification (Taste and Smell)	Sensory Quality Evaluation
64	13.06	3.06	Low score
138	11.40	3.30	
313	14.30	3.60	
877	13.90	3.65	
63	14.72	3.72	
125	14.07	3.86	
628	14.70	4.25	High score
748	15.64	4.45	
679	17.27	5.14	
110	17.45	5.25	

**Table 3:** Tentatively identified compounds organized by different families, in bold, and their relative average areas ± standard deviation (n = 3) for each cheese sample. * Compounds highlighted in Figure 1. Different letters within each row indicate significant differences between samples per compound/family, at the *p* < 0.05 level. The average and standard error of the mean (SEM) for the groups—low score, n = 18 vs. high score, n = 12—are presented. Differences between groups (low and high score cheeses, highlighted with different background color), *p* < 0.05, are indicated by different letters.

[#] Tentatively Identified Compounds	Low Score							High Score				
	63	64	125	138	313	877	Average ± SEM	110	628	679	748	Average ± SEM
[#3 *] Ethanol	13.26 ± 0.24 ^bc^	11.95 ± 0.30 ^e^	15.29 ± 0.16 ^bc^	15.38 ± 0.84 ^b^	14.84 ± 0.50 ^bcd^	14.15 ± 1.02 ^bcd^	14.14 ± 0.32 ^a^	11.86 ± 0.14 ^e^	19.51 ± 0.59 ^a^	13.01 ± 0.71 ^de^	14.66 ± 0.21 ^bcd^	14.76 ± 0.89 ^a^
[#8 *] 2-Butanol	10.74 ± 1.37 ^a^	4.06 ± 030 ^d^	4.29 ± 0.38 ^d^	4.52 ± 0.61 ^cd^	11.31 ± 0.41 ^a^	6.59 ± 0.33 ^bc^	6.92 ± 0.75 ^a^	4.57 ± 0.20 ^cd^	7.91 ± 0.44 ^b^	4.18 ± 0.17 ^d^	4.25 ± 0.27 ^d^	5.23 ± 0.47 ^a^
[#14] Isobutanol	0.17 ± 0.02 ^a^	0.09 ± 0.02 ^ab^	0.12 ± 0.01 ^abc^	0.13 ± 0.03 ^abc^	0.16 ± 0.01	0.14 ± 0.02 ^ab^	0.14 ± 0.01 ^a^	0.16 ± 0.02 ^a^	0.08 ± 0.02 ^c^	0.15 ± 0.01 ^ab^	0.12 ± 0.01 ^abc^	0.13 ± 0.01 ^a^
[#16] 2-Pentanol	0.22 ± 0.16 ^a^	0.05 ± 0.02 ^a^	0.08 ± 0.00 ^a^	0.06 ± 0.01 ^a^	0.06 ± 0.01 ^a^	0.04 ± 0.01 ^a^	0.08 ± 0.02 ^a^	0.11 ± 0.01 ^a^	0.07 ± 0.01 ^a^	0.04 ± 0.03 ^a^	0.05 ± 0.01 ^a^	0.07 ± 0.01 ^a^
[#18] 1-Butanol	0.09 ± 0.02 ^cd^	0.18 ± 0.04 ^ab^	0.15 ± 0.04 ^abc^	0.22 ± 0.02 ^a^	0.06 ± 0.01 ^d^	0.12 ± 0.01 ^bcd^	0.14 ± 0.01 ^a^	0.15 ± 0.02 ^abc^	0.12 ± 0.01 ^bcd^	0.15 ± 0.02 ^abc^	0.14 ± 0.01 ^abc^	0.14 ± 0.01 ^a^
[#26 *] Isoamyl alcohol	5.25 ± 0.10 ^bc^	2.39 ± 0.20 ^e^	5.22 ± 0.20 ^bc^	6.56 ± 1.15 ^ab^	6.00 ± 0.21 ^b^	4.09 ± 0.17 ^cd^	4.92 ± 0.34 ^a^	7.75 ± 0.18 ^a^	2.87 ± 0.49 ^de^	5.64 ± 0.10 ^bc^	4.10 ± 0.23 ^cd^	5.09 ± 0.55 ^a^
[#34] 1H,1H,2H,2H-Perfluorooctan-1-ol isomer	0.14 ± 0.11 ^bcd^	0.10 ± 0.04 ^bcd^	0.09 ± 0.06 ^bcd^	0.30 ± 0.03 ^ab^	0.05 ± 0.02 ^cd^	0.33 ± 0.04 ^ab^	0.17 ± 0.03 ^a^	0.05 ± 0.02 ^d^	0.29 ± 0.11 ^abc^	0.02 ± 0.01 ^d^	0.48 ± 0.10 ^a^	0.21 ± 0.06 ^a^
[#44] 2-Heptanol	0.46 ± 0.07 ^a^	0.14 ± 0.02 ^cd^	0.16 ± 0.02 ^cd^	0.12 ± 0.02 ^cd^	0.06 ± 0.01 ^d^	0.14 ± 0.03 ^cd^	0.18 ± 0.03 ^a^	0.38 ± 0.04 ^ab^	0.26 ± 0.07 ^bc^	0.23 ± 0.08 ^bcd^	0.09 ± 0.01 ^d^	0.24 ± 0.03 ^a^
[#48] 1H,1H,2H,2H-Perfluorooctan-1-ol isomer	0.01 ± 0.01 ^c^	0.01 ± 0.00 ^c^	0.01 ± 0.01 ^c^	0.03 ± 0.01 ^bc^	0.00 ± 0.01 ^c^	0.06 ± 0.02 ^ab^	0.02 ± 0.01 ^a^	<0.01 ^c^	0.02 ± 0.01 ^c^	<0.01 ^c^	0.10 ± 0.02 ^a^	0.03 ± 0.01 ^a^
[#59 *] 2-Butoxyethanol	0.29 ± 0.01 ^b^	0.05 ± 0.00 ^c^	0.07 ± 0.02 ^c^	0.08 ± 0.02 ^c^	0.84 ± 0.04 ^a^	0.28 ± 0.07 ^b^	0.27 ± 0.07 ^a^	0.37 ± 0.03 ^b^	0.05 ± 0.01 ^c^	0.29 ± 0.01 ^b^	0.31 ± 0.04 ^b^	0.26 ± 0.04 ^a^
[#68] 2-Ethylhexanol	0.02 ± 0.01 ^c^	0.06 ± 0.02 ^abc^	0.11 ± 0.02 ^ab^	0.05 ± 0.01 ^abc^	0.05 ± 0.01 ^bc^	0.08 ± 0.02 ^abc^	0.06 ± 0.01 ^a^	0.05 ± 0.02 ^abc^	0.02 ± 0.01 ^c^	0.07 ± 0.04 ^abc^	0.13 ± 0.04 ^a^	0.07 ± 0.01 ^a^
[#75] 2,3-Butanediol	0.03 ± 0.01 ^de^	0.01 ± 0.00 ^f^	0.03 ± 0.00 ^e^	0.03 ± 0.01 ^e^	0.05 ± 0.01 ^cd^	0.01 ± 0.00 ^f^	0.03 ± 0.00 ^b^	0.09 ± 0.01 ^b^	0.05 ± 0.00 ^c^	0.17 ± 0.01 ^a^	0.01 ± 0.00 ^f^	0.08 ± 0.02 ^a^
[#76] trans,trans-2,4-Hexadien-1-ol	<0.01 ^c^	<0.01 ^c^	<0.01 ^c^	0.03 ± 0.01 ^a^	<0.01 ^c^	<0.01 ^c^	0.01 ± 0.00 ^a^	<0.01 ^c^	0.02 ± 0.02 ^ab^	<0.01 ^c^	0.01 ± 0.01 ^bc^	0.01 ± 0.00 ^a^
[#80] trans-2-octenol	<0.01 ^d^	<0.01 ^d^	<0.01 ^d^	0.02 ± 0.01 ^bc^	0.01 ± 0.00 ^c^	0.01 ± 0.00 ^c^	0.01 ± 0.00 ^b^	0.04 ± 0.01 ^a^	0.01 ± 0.00 ^c^	0.02 ± 0.00 ^b^	<0.01 ^d^	0.02 ± 0.00 ^a^
[#84] Furfuryl alcohol	<0.01 ^c^	<0.01 ^c^	0.01 ± 0.00 ^abc^	0.02 ± 0.01 ^ab^	0.02 ± 0.00 ^a^	0.00 ± 0.01 ^bc^	0.01 ± 0.00 ^a^	<0.01 ^c^	0.01 ± 0.00 abc	0.01 ± 0.00 ^abc^	0.01 ± 0.00 ^abc^	0.01 ± 0.00 ^a^
[#90] Methionol	<0.01 ^c^	0.01 ± 0.00 ^c^	0.03 ± 0.01 ^bc^	0.05 ± 0.02 ^b^	0.01 ± 0.00 ^c^	0.02 ± 0.00 ^c^	0.02 ± 0.00 ^b^	0.09 ± 0.01 ^a^	<0.01 ^c^	0.11 ± 0.01 ^a^	0.01 ± 0.01 ^c^	0.06 ± 0.01 ^a^
[#97] 1-Decanol	0.01 ± 0.00 ^d^	0.08 ± 0.01 ^ab^	0.02 ± 0.01 ^cd^	0.00 ± 0.01 ^d^	0.01 ± 0.01 ^d^	0.10 ± 0.02 ^a^	0.04 ± 0.01 ^a^	0.02 ± 0.02 ^cd^	0.01 ± 0.00 ^d^	0.01 ± 0.01 ^d^	0.05 ± 0.01 ^bc^	0.02 ± 0.01 ^a^
[#104] 1-Phenyl-2-propanol	<0.01 ^b^	<0.01 ^b^	0.01 ± 0.01 ^a^	<0.01 ^b^	<0.01 ^b^	0.01 ± 0.00 ^a^	<0.01 ^a^	<0.01 ^b^	0.01 ± 0.00 ^a^	<0.01 ^b^	<0.01 ^b^	<0.01 ^a^
[#109 *] Benzyl alcohol	0.18 ± 0.01 ^bc^	0.12 ± 0.01 ^cd^	0.18 ± 0.02 ^bc^	0.04 ± 0.02 ^d^	0.36 ± 0.01 ^a^	0.17 ± 0.04 ^bcd^	0.18 ± 0.02 ^a^	0.29 ± 0.09 ^ab^	0.04 ± 0.00 ^d^	0.16 ± 0.05 ^bcd^	0.10 ± 0.01 ^cd^	0.15 ± 0.03 ^a^
[#115 *] Phenethyl alcohol	1.40 ± 0.02 ^b^	0.48 ± 0.05 ^ef^	1.10 ± 0.05 ^bcd^	1.93 ± 0.27 ^a^	0.98 ± 0.07 ^cd^	0.77 ± 0.08 ^de^	1.11 ± 0.12 ^a^	1.22 ± 0.08 ^bc^	0.28 ± 0.02 ^f^	1.03 ± 0.09 ^bcd^	0.53 ± 0.05 ^ef^	0.77 ± 0.11 ^a^
[#120] 1-Dodecanol	0.02 ± 0.01 ^a^	<0.01 ^b^	<0.01 ^b^	0.01 ± 0.01 ^a^	0.01 ± 0.00 ^ab^	<0.01 ^b^	0.01 ± 0.00 ^a^	<0.01 ^b^	0.01 ± 0.01 ^ab^	<0.01 ^b^	<0.01 ^b^	<0.01 ^b^
**Alcohol**	**32.28 ± 1.49 ^ab^**	**19.78 ± 0.60 ^e^**	**26.96 ± 0.34 ^cd^**	**29.59 ± 2.00 ^bc^**	**34.88 ± 0.60 ^a^**	**27.12 ± 0.98 ^cd^**	**28.44 ± 1.18 ^a^**	**27.21 ± 0.38 ^cd^**	**31.64 ± 0.51 ^ab^**	**25.28 ± 0.76 ^d^**	**25.15 ± 0.60 ^d^**	**27.32 ± 0.80 ^a^**
[#12 *] Hexanal	<0.01 ^b^	0.01 ± 0.01 ^ab^	<0.01 ^b^	0.01 ± 0.00 ^ab^	<0.01 ^b^	0.01 ± 0.01 ^ab^	<0.01 ^a^	<0.01 ^b^	0.02 ± 0.01 ^a^	<0.01 ^b^	0.01 ± 0.00 ^ab^	0.01 ± 0.00 ^a^
[#58] Nonanal	0.04 ± 0.03 ^a^	0.05 ± 0.01 ^a^	0.07 ± 0.03 ^a^	0.06 ± 0.03 ^a^	0.03 ± 0.01 ^a^	0.04 ± 0.01 ^a^	0.05 ± 0.01 ^a^	0.01 ± 0.01 ^a^	0.07 ± 0.06 ^a^	0.02 ± 0.01 ^a^	0.04 ± 0.03 ^a^	0.04 ± 0.01 ^a^
[#61] trans-2-Octenal	<0.01 ^a^	0.01 ± 0.00 ^a^	<0.01 ^a^	<0.01 ^a^	<0.01 ^a^	<0.01 ^a^	<0.01 ^a^	<0.01 ^a^	<0.01 ^a^	<0.01 ^a^	0.01 ± 0.01 ^a^	<0.01 ^a^
[#70] Benzaldehyde	0.32 ± 0.07 ^a^	0.27 ± 0.04 ^ab^	0.16 ± 0.07 ^bcd^	0.03 ± 0.01 ^d^	0.08 ± 0.01 ^cd^	0.24 ± 0.04 ^abc^	0.19 ± 0.03 ^a^	0.16 ± 0.05 ^bcd^	0.23 ± 0.03 ^abc^	0.06 ± 0.04 ^d^	0.14 ± 0.03 ^bcd^	0.15 ± 0.02 ^a^
**Aldehyde**	**0.37 ± 0.05 ^a^**	**0.34 ± 0.03 ^a^**	**0.23 ± 0.06 ^abc^**	**0.10 ± 0.03 ^c^**	**0.11 ± 0.02 ^c^**	**0.28 ± 0.04 ^ab^**	**0.24 ± 0.03 ^a^**	**0.17 ± 0.04 ^bc^**	**0.32 ± 0.05 ^ab^**	**0.08 ± 0.04 ^c^**	**0.20 ± 0.06 ^abc^**	**0.19 ± 0.03 ^a^**
[#6] Decane	0.14 ± 0.04 ^ab^	0.10 ± 0.04 ^ab^	0.16 ± 0.07 ^a^	0.05 ± 0.01 ^ab^	0.02 ± 0.01 ^b^	0.02 ± 0.02 ^b^	0.08 ± 0.01 ^a^	0.03 ± 0.02 ^b^	0.07 ± 0.02 ^ab^	0.04 ± 0.03 ^ab^	0.03 ± 0.03 ^b^	0.05 ± 0.01 ^a^
[#10] 3-Methyldecane	0.02 ± 0.01 ^abc^	0.03 ± 0.01 ^ab^	0.04 ± 0.01 ^a^	0.01 ± 0.01 ^abc^	0.01 ± 0.01 ^bc^	0.01 ± 0.00 ^bc^	0.02 ± 0.00 ^a^	<0.01 ^c^	0.01 ± 0.01 ^abc^	0.01 ± 0.01 ^bc^	0.01 ± 0.01 ^bc^	0.01 ± 0.00 ^b^
[#13] Undecane	0.03 ± 0.01 ^a^	0.03 ± 0.01 ^a^	0.04 ± 0.02 ^a^	0.01 ± 0.01 ^a^	0.04 ± 0.01 ^a^	0.03 ± 0.02 ^a^	0.03 ± 0.00 ^a^	0.01 ± 0.01 ^a^	0.02 ± 0.01 ^a^	0.01 ± 0.01 ^a^	0.01 ± 0.01 ^a^	0.01 ± 0.00 ^b^
**Alkane**	**0.20 ± 0.05 ^ab^**	**0.16 ± 0.06 ^ab^**	**0.23 ± 0.10 ^a^**	**0.07 ± 0.02 ^ab^**	**0.07 ± 0.01 ^ab^**	**0.06 ± 0.03 ^b^**	**0.13 ± 0.02 ^a^**	**0.04 ± 0.03 ^b^**	**0.10 ± 0.03 ^ab^**	**0.06 ± 0.04 ^ab^**	**0.05 ± 0.03 ^b^**	**0.07 ± 0.01 ^b^**
[#96] Acetamide	0.02 ± 0.01 ^a^	0.01 ± 0.00 ^a^	<0.01 ^a^	0.01 ± 0.00 ^a^	<0.01 ^a^	<0.01 ^a^	0.01 ± 0.00 ^a^	0.01 ± 0.00 ^a^	0.01 ± 0.01 ^a^	0.01 ± 0.00 ^a^	<0.01 ^a^	0.01 ± 0.00 ^a^
[#98] Formamide	<0.01 ^b^	<0.01 ^b^	0.01 ± 0.00 ^ab^	<0.01 ^b^	<0.01 ^b^	<0.01 ^b^	<0.01 ^a^	<0.01 ^b^	<0.01 ^b^	0.01 ± 0.01 ^a^	<0.01 ^b^	<0.01 ^a^
**Amide**	**0.02 ± 0.01 ^a^**	**0.01 ± 0.00 ^a^**	**0.01 ± 0.01 ^a^**	**0.01 ± 0.00 ^a^**	**<0.01 ^a^**	**<0.01 ^a^**	**0.01 ± 0.00 ^a^**	**0.01 ± 0.00 ^a^**	**0.01 ± 0.01 ^a^**	**0.02 ± 0.01 ^a^**	**<0.01 ^a^**	**0.01 ± 0.00 ^a^**
[#54] 2-Ethylbutyric acid	<0.01 ^b^	<0.01 ^b^	<0.01 ^b^	<0.01 ^b^	<0.01 ^b^	0.02 ± 0.01 ^a^	<0.01 ^a^	<0.01 ^b^	<0.01 ^b^	<0.01 ^b^	<0.01 ^b^	<0.01 ^a^
[#64 *] Acetic acid	5.98 ± 0.22 ^c^	2.82 ± 0.16 ^f^	5.58 ± 0.17 ^c^	7.38 ± 0.35 ^b^	4.16 ± 0.22 ^e^	4.44 ± 0.22 ^de^	5.06 ± 0.36 ^b^	7.18 ± 0.32 ^b^	7.99 ± 0.19 ^ab^	8.45 ± 0.27 ^a^	5.18 ± 0.14 ^cd^	7.20 ± 0.38 ^a^
[#72 *] Propanoic acid	0.72 ± 0.11 ^e^	7.17 ± 0.12 ^a^	2.64 ± 0.06 ^cd^	1.16 ± 0.08 ^e^	4.60 ± 0.29 ^b^	2.29 ± 0.10 ^d^	3.10 ± 0.53 ^a^	1.13 ± 0.09 ^e^	0.48 ± 0.08 ^f^	0.78 ± 0.03 ^ef^	2.91 ± 0.13 ^c^	1.33 ± 0.29 ^b^
[#74 *] Isobutyric acid	0.32 ± 0.03 ^g^	1.92 ± 0.08 ^d^	2.77 ± 0.05 ^bc^	1.23 ± 0.12 ^e^	0.77 ± 0.02 ^f^	3.02 ± 0.16 ^ab^	1.67 ± 0.24 ^a^	3.24 ± 0.17 ^a^	0.76 ± 0.04 ^f^	2.46 ± 0.13 ^c^	2.41 ± 0.02 ^c^	2.22 ± 0.27 ^a^
[#81 *] Butanoic acid	4.83 ± 0.19 ^f^	16.49 ± 0.47 ^a^	12.06 ± 0.07 ^b^	7.73 ± 0.21 ^cd^	3.69 ± 0.19 ^b^	6.84 ± 0.23 ^de^	8.61 ± 1.07 ^a^	6.41 ± 0.10 ^e^	4.43 ± 0.14 ^fg^	8.24 ± 0.17 ^c^	11.28 ± 0.41 ^b^	7.59 ± 0.76 ^a^
[#86 *] Isovaleric acid	1.83 ± 0.05 ^f^	6.55 ± 0.11 ^d^	8.75 ± 0.19 ^b^	6.66 ± 0.30 ^d^	2.67 ± 0.23 ^f^	7.55 ± 0.07 ^c^	5.67 ± 0.61 ^b^	11.45 ± 0.46 ^a^	3.95 ± 0.20 ^e^	8.95 ± 0.28 ^b^	7.14 ± 0.16 ^cd^	7.87 ± 0.83 ^a^
[#93 *] Valeric acid	0.24 ± 0.07 ^b^	1.45 ± 0.23 ^a^	0.30 ± 0.02 ^b^	0.25 ± 0.03 ^b^	0.11 ± 0.01 ^b^	0.30 ± 0.01 ^b^	0.44 ± 0.11 ^a^	0.28 ± 0.02 ^b^	0.20 ± 0.03 ^b^	0.37 ± 0.01 ^b^	0.37 ± 0.06 ^b^	0.30 ± 0.02 ^a^
[#102] 4-Methylvaleric acid	<0.01 ^d^	0.19 ± 0.02 ^a^	0.11 ± 0.01 ^b^	0.04 ± 0.01 ^c^	<0.01 ^d^	0.05 ± 0.01 ^c^	0.07 ± 0.02 ^a^	0.05 ± 0.00 ^c^	0.03 ± 0.00 ^cd^	0.05 ± 0.00 ^c^	0.10 ± 0.02 ^b^	0.06 ± 0.01 ^a^
[#105 *] Caproic acid	6.78 ± 0.45 ^e^	10.23 ± 0.46 ^a^	7.59 ± 0.18 ^bcde^	8.46 ± 0.90 ^bcd^	5.04 ± 0.16 ^f^	7.41 ± 0.23 ^cde^	7.58 ± 0.39 ^a^	7.00 ± 0.29 ^de^	6.45 ± 0.08 ^ef^	8.78 ± 0.35 ^abc^	9.01 ± 0.30 ^ab^	7.81 ± 0.34 ^a^
[#113] 5-Hexenoic acid	0.01 ± 0.01 ^d^	0.16 ± 0.02 ^a^	0.09 ± 0.00 ^b^	0.04 ± 0.02 ^cd^	0.01 ± 0.00 ^cd^	0.04 ± 0.01 ^c^	0.06 ± 0.01 ^a^	0.09 ± 0.01 ^b^	0.02 ± 0.01 ^cd^	0.11 ± 0.00 ^b^	0.08 ± 0.01 ^b^	0.08 ± 0.01 ^a^
[#114] 4-Hexenoic acid	<0.01 ^b^	<0.01 ^b^	<0.01 ^b^	<0.01 ^b^	0.73 ± 0.02 ^a^	<0.01 ^b^	0.12 ± 0.07 ^a^	<0.01 ^b^	<0.01 ^b^	<0.01 ^b^	<0.01 ^b^	<0.01 ^a^
[#116] 2-Methylhexanoic acid	0.01 ± 0.00 ^c^	0.19 ± 0.03 ^a^	0.07 ± 0.01 ^b^	0.02 ± 0.01 ^c^	0.01 ± 0.01 ^c^	<0.01 ^c^	0.05 ± 0.02 ^a^	0.03 ± 0.02 ^bc^	<0.01 ^c^	0.02 ± 0.01 ^bc^	0.03 ± 0.02 ^bc^	0.02 ± 0.00 ^a^
[#118 *] Heptanoic acid	0.38 ± 0.05 ^b^	0.37 ± 0.04 ^b^	0.21 ± 0.01 ^c^	0.33 ± 0.03 ^bc^	0.29 ± 0.02 ^bc^	0.63 ± 0.02 ^a^	0.37 ± 0.03 ^a^	0.24 ± 0.03 ^c^	0.27 ± 0.01 b^c^	0.30 ± 0.06 ^bc^	0.39 ± 0.03 ^b^	0.30 ± 0.02 ^a^
[#124] Caprylic acid	4.62 ± 0.31 ^ab^	4.83 ± 0.40 ^ab^	3.64 ± 0.11 ^bc^	5.25 ± 0.87 ^a^	3.61 ± 0.04 ^bc^	4.47 ± 0.08 ^ab^	4.40 ± 0.17 ^a^	4.13 ± 0.11 ^abc^	3.04 ± 0.07 ^c^	4.43 ± 0.42 ^ab^	4.50 ± 0.14 ^ab^	4.03 ± 0.19 ^a^
[#128] Sorbic acid isomer	<0.01 ^b^	<0.01 ^b^	0.01 ± 0.01 ^b^	0.08 ± 0.02 ^a^	<0.01 ^b^	<0.01 ^b^	0.02 ± 0.01 ^a^	0.01 ± 0.00 ^b^	<0.01 ^b^	<0.01 ^b^	<0.01 ^b^	<0.01 ^a^
[#129] Sorbic acid isomer	0.01 ± 0.01 ^b^	0.02 ± 0.00 ^b^	0.01 ± 0.01 ^b^	1.72 ± 0.24 ^a^	0.01 ± 0.00 ^b^	0.01 ± 0.00 ^b^	0.30 ± 0.16 ^a^	0.01 ± 0.01 ^b^	0.01 ± 0.01 ^b^	<0.01 ^b^	0.04 ± 0.04 ^b^	0.01 ± 0.01 ^a^
[#130 *] Nonanoic acid	1.06 ± 0.06 ^a^	0.77 ± 0.24 ^ab^	0.25 ± 0.07 ^b^	0.59 ± 0.33 ^ab^	0.61 ± 0.14 ^ab^	0.63 ± 0.15 ^ab^	0.65 ± 0.07 ^a^	1.15 ± 0.18 ^a^	0.16 ± 0.02 ^b^	0.69 ± 0.16 ^ab^	0.33 ± 0.13 ^b^	0.58 ± 0.12 ^a^
[#134 *] n-Capric acid	4.57 ± 0.44 ^d^	3.06 ± 0.23 ^bcd^	2.21 ± 0.06 ^cd^	4.10 ± 1.02 ^ab^	3.35 ± 0.05 ^abc^	3.84 ± 0.08 ^ab^	3.52 ± 0.21 ^a^	2.79 ± 0.07 ^bcd^	1.84 ± 0.06 ^d^	3.18 ± 0.19 ^bcd^	2.96 ± 0.04 ^bcd^	2.69 ± 0.16 ^b^
[#135] Caproleic acid	0.28 ± 0.03 ^a^	0.10 ± 0.01 ^c^	0.08 ± 0.02 ^c^	0.15 ± 0.05 ^b^	0.13 ± 0.01 ^c^	0.15 ± 0.01 ^b^	0.15 ± 0.02 ^a^	0.10 ± 0.00 ^c^	0.05 ± 0.01 ^c^	0.11 ± 0.02 ^c^	0.10 ± 0.01 ^c^	0.09 ± 0.01 ^b^
[#136] Undecanoic acid	0.05 ± 0.01 ^ab^	0.02 ± 0.00 ^c^	0.02 ± 0.01 ^c^	0.02 ± 0.01 ^c^	0.04 ± 0.00 ^b^	0.06 ± 0.01 ^a^	0.04 ± 0.00 ^a^	0.02 ± 0.00 ^c^	0.01 ± 0.01 ^c^	0.02 ± 0.00 ^c^	0.02 ± 0.00 ^c^	0.02 ± 0.00 ^b^
[#140] Benzoic acid	0.06 ± 0.01 ^bc^	0.07 ± 0.01 ^b^	0.03 ± 0.00 ^d^	0.08 ± 0.01 ^ab^	0.04 ± 0.01 ^cd^	0.06 ± 0.01 ^b^	0.06 ± 0.00 ^a^	0.07 ± 0.01 ^b^	<0.01 ^e^	0.11 ± 0.01 ^a^	0.06 ± 0.00 ^bc^	0.06 ± 0.01 ^a^
[#141 *] Lauric acid	0.44 ± 0.08 ^a^	0.22 ± 0.04 ^bcd^	0.15 ± 0.00 ^cd^	0.23 ± 0.06 ^bcd^	0.31 ± 0.03 ^ab^	0.43 ± 0.02 ^a^	0.30 ± 0.03 ^a^	0.17 ± 0.01 ^bcd^	0.13 ± 0.02 ^d^	0.20 ± 0.03 ^bcd^	0.27 ± 0.01 ^bc^	0.19 ± 0.02 ^b^
[#143] Phenylpropanedioic acid	<0.01 ^b^	<0.01 ^b^	0.07 ± 0.01 ^a^	<0.01 ^b^	<0.01 ^b^	0.07 ± 0.01 ^a^	0.02 ± 0.01 ^a^	<0.01 ^b^	<0.01 ^b^	<0.01 ^b^	0.09 ± 0.03 ^a^	0.02 ± 0.01 ^a^
[#144] Myristic acid	0.12 ± 0.04 ^ab^	0.14 ± 0.01 ^ab^	0.12 ± 0.04 ^ab^	0.19 ± 0.01 ^ab^	0.14 ± 0.02 ^ab^	0.18 ± 0.02 ^ab^	0.15 ± 0.01 ^a^	0.09 ± 0.03 ^b^	0.11 ± 0.03 ^ab^	0.08 ± 0.03 ^b^	0.18 ± 0.01 ^ab^	0.12 ± 0.01 ^b^
**Carboxylic acid**	**32.32 ± 1.96 ^d^**	**56.85 ± 1.19 ^a^**	**46.77 ± 0.27 ^bc^**	**45.72 ± 2.60 ^bc^**	**30.32 ± 1.18 ^d^**	**42.54 ± 0.47 ^c^**	**42.42 ± 2.20 ^a^**	**45.63 ± 0.78 ^bc^**	**29.94 ± 0.33 ^d^**	**47.33 ± 0.61 ^b^**	**47.46 ± 1.08 ^b^**	**42.59 ± 2.22 ^a^**
[#25] n-Propylbenzene	<0.01 ^a^	<0.01 ^a^	<0.01 ^a^	<0.01 ^a^	<0.01 ^a^	<0.01 ^a^	<0.01 ^a^	<0.01 ^a^	<0.01 ^a^	<0.01 ^a^	<0.01 ^a^	<0.01 ^a^
[#30] Sec-butylbenzene	<0.01 ^a^	<0.01 ^a^	<0.01 ^a^	<0.01 ^a^	<0.01 ^a^	<0.01 ^a^	<0.01 ^a^	<0.01 ^a^	<0.01 ^a^	<0.01 ^a^	<0.01 ^a^	<0.01 ^a^
[#31] Styrene	0.02 ± 0.01 ^ab^	0.03 ± 0.00 ^ab^	0.02 ± 0.00 ^ab^	0.06 ± 0.02 ^a^	0.01 ± 0.01 ^b^	0.03 ± 0.01 ^ab^	0.03 ± 0.00 ^a^	0.03 ± 0.02 ^ab^	0.01 ± 0.01 ^b^	0.04 ± 0.02 ^ab^	0.05 ± 0.01 ^ab^	0.03 ± 0.01 ^a^
[#32] Cumene	0.01 ± 0.01 ^a^	<0.01 ^a^	0.01 ± 0.01 ^a^	<0.01 ^a^	<0.01 ^a^	<0.01 ^a^	<0.01 ^a^	<0.01 ^a^	<0.01 ^a^	<0.01 ^a^	<0.01 ^a^	<0.01 ^a^
[#36] 1,2,4-Trimethylbenzene	0.04 ± 0.01 ^a^	0.03 ± 0.00 ^a^	0.04 ± 0.02 ^a^	0.01 ± 0.01 ^a^	0.01 ± 0.00 ^a^	0.01 ± 0.00 ^a^	0.02 ± 0.00 ^a^	0.01 ± 0.00 ^a^	0.03 ± 0.01 ^a^	0.02 ± 0.01 ^a^	0.02 ± 0.01 ^a^	0.02 ± 0.00 ^a^
[#39] *p*-Diethylbenzene	<0.01 ^a^	<0.01 ^a^	<0.01 ^a^	<0.01 ^a^	<0.01 ^a^	<0.01 ^a^	<0.01 ^a^	<0.01 ^a^	<0.01 ^a^	0.01 ± 0.02 ^a^	<0.01 ^a^	<0.01 ^a^
[#41] 1,3-Diethylbenzene	<0.01 ^a^	<0.01 ^a^	<0.01 ^a^	<0.01 ^a^	<0.01 ^a^	<0.01 ^a^	<0.01 ^a^	<0.01 ^a^	<0.01 ^a^	<0.01 ^a^	<0.01 ^a^	<0.01 ^a^
[#42] n-Butylbenzene	<0.01 ^a^	<0.01 ^a^	<0.01 ^a^	<0.01 ^a^	<0.01 ^a^	<0.01 ^a^	<0.01 ^a^	<0.01 ^a^	<0.01 ^a^	<0.01 ^a^	<0.01 ^a^	<0.01 ^a^
[#49] 2-Ethyl-*p*-xylene	0.01 ± 0.01 ^a^	<0.01 ^a^	0.01 ± 0.01 ^a^	<0.01 ^a^	<0.01 ^a^	<0.01 ^a^	<0.01 ^a^	<0.01 ^a^	<0.01 ^a^	<0.01 ^a^	<0.01 ^a^	<0.01 ^a^
[#52] 4-Ethyl-o-xylene	<0.01 ^a^	<0.01 ^a^	0.01 ± 0.01 ^a^	<0.01 ^a^	<0.01 ^a^	<0.01 ^a^	<0.01 ^a^	<0.01 ^a^	<0.01 ^a^	<0.01 ^a^	<0.01 ^a^	<0.01 ^a^
[#95] *p*-Dimethoxybenzene	<0.01 ^a^	<0.01 ^a^	<0.01 ^a^	<0.01 ^a^	<0.01 ^a^	<0.01 ^a^	<0.01 ^a^	<0.01 ^a^	<0.01 ^a^	<0.01 ^a^	<0.01 ^a^	<0.01 ^a^
[#121] Phenol	0.03 ± 0.01 ^b^	0.35 ± 0.13 ^ab^	0.09 ± 0.03 ^b^	0.10 ± 0.04 ^b^	0.02 ± 0.00 ^b^	0.11 ± 0.03 ^b^	0.12 ± 0.03 ^a^	0.04 ± 0.00 ^b^	0.02 ± 0.01 ^b^	0.03 ± 0.01 ^b^	0.66 ± 0.28 ^a^	0.19 ± 0.09 ^a^
[#125] *p*-Cresol	0.01 ± 0.00 ^bc^	0.02 ± 0.01 ^a^	0.01 ± 0.00 ^bc^	<0.01 ^d^	0.01 ± 0.00 ^bc^	0.01 ± 0.00 ^bc^	0.01 ± 0.00 ^a^	0.01 ± 0.01 ^b^	0.01 ± 0.00 ^bc^	<0.01 ^cd^	<0.01 ^d^	0.01 ± 0.00 ^a^
[#126] m-Cresol	<0.01 ^d^	0.03 ± 0.01 ^a^	0.02 ± 0.01 ^bc^	<0.01 ^d^	0.01 ± 0.00 ^cd^	0.03 ± 0.01 ^ab^	0.01 ± 0.00 ^a^	0.02 ± 0.01 ^bc^	<0.01 ^d^	0.01 ± 0.01 ^cd^	<0.01 ^d^	0.01 ± 0.00 ^a^
[#139] Indole	<0.01 ^b^	0.10 ± 0.06 ^a^	0.02 ± 0.01 ^b^	<0.01 ^b^	<0.01 ^b^	<0.01 ^b^	0.02 ± 0.01 ^a^	<0.01 ^b^	<0.01 ^b^	<0.01 ^b^	<0.01 ^b^	<0.01 ^a^
[#142] 3-Methylindole	<0.01 ^a^	<0.01 ^a^	<0.01 ^a^	<0.01 ^a^	<0.01 ^a^	<0.01 ^a^	<0.01 ^a^	<0.01 ^a^	<0.01 ^a^	<0.01 ^a^	<0.01 ^a^	<0.01 ^a^
**Cyclic Hydrocarbon**	**0.13 ± 0.02 ^c^**	**0.57 ± 0.18 ^ab^**	**0.23 ± 0.04 ^bc^**	**0.17 ± 0.06 ^bc^**	**0.07 ± 0.01 ^c^**	**0.19 ± 0.03 ^bc^**	**0.23 ± 0.04 ^a^**	**0.11 ± 0.03 ^c^**	**0.07 ± 0.02 ^c^**	**0.12 ± 0.03 ^c^**	**0.73 ± 0.27 ^a^**	**0.26 ± 0.09 ^a^**
[#1] Ethyl acetate	0.02 ± 0.01 ^b^	<0.01 ^c^	0.05 ± 0.01 ^a^	<0.01 ^c^	<0.01 ^c^	<0.01 ^c^	0.01 ± 0.00 ^a^	<0.01 ^c^	0.03 ± 0.00 ^b^	0.02 ± 0.00 ^b^	<0.01 ^c^	0.01 ± 0.00 ^a^
[#5] Sec-butyl acetate	0.02 ± 0.02 ^a^	<0.01 ^a^	<0.01 ^a^	<0.01 ^a^	0.02 ± 0.01 ^a^	<0.01 ^a^	0.01 ± 0.00 ^a^	<0.01 ^a^	<0.01 ^a^	<0.01 ^a^	<0.01 ^a^	<0.01 ^a^
[#9 *] Ethyl butyrate	7.97 ± 0.67 ^ab^	4.74 ± 0.20 ^cd^	3.58 ± 0.18 ^d^	3.90 ± 0.98 ^d^	9.06 ± 0.92 ^a^	6.25 ± 0.28 ^bc^	5.92 ± 0.51 ^a^	4.61 ± 0.14 ^cd^	6.29 ± 0.34 ^bc^	4.64 ± 0.19 ^cd^	4.67 ± 0.43 ^cd^	5.05 ± 0.23 ^a^
[#11 *] Ethyl isovalerate	0.04 ± 0.01 ^b^	0.17 ± 0.05 ^a^	0.12 ± 0.05 ^ab^	0.12 ± 0.04 ^ab^	0.03 ± 0.01 ^b^	0.09 ± 0.01 ^ab^	0.10 ± 0.01 ^a^	0.10 ± 0.01 ^ab^	0.11 ± 0.02 ^ab^	0.08 ± 0.02 ^ab^	0.05 ± 0.02 ^b^	0.09 ± 0.01 ^a^
[#15] Isoamyl acetate	0.79 ± 0.09 ^ab^	0.22 ± 0.02 ^ef^	0.36 ± 0.01 ^def^	0.47 ± 0.08 ^cde^	0.58 ± 0.04 ^bcd^	0.21 ± 0.01 ^f^	0.44 ± 0.05 ^a^	0.71 ± 0.09 ^bc^	0.28 ± 0.01 ^ef^	0.96 ± 0.14 ^a^	0.24 ± 0.05 ^ef^	0.55 ± 0.09 ^a^
[#20] Isobutyl butyrate]	0.01 ± 0.01 ^ab^	0.01 ± 0.00 ^ab^	0.01 ± 0.01 ^ab^	<0.01 ^b^	0.01 ± 0.00 ^ab^	<0.01 ^ab^	0.01 ± 0.00 ^a^	0.02 ± 0.01 ^a^	<0.01 ^b^	0.01 ± 0.01 ^ab^	<0.01 ^b^	0.01 ± 0.00 ^a^
[#21] Alkyl butyrate	<0.01 ^b^	<0.01 ^b^	<0.01 ^b^	<0.01 ^b^	<0.01 ^b^	<0.01 ^b^	<0.01 ^a^	0.01 ± 0.01 ^ab^	<0.01 ^b^	0.02 ± 0.00 ^a^	<0.01 ^b^	0.01 ± 0.00 ^a^
[#27] 1-Methoxy-2-propyl acetate	<0.01 ^b^	<0.01 ^b^	0.02 ± 0.01 ^a^	0.01 ± 0.01 ^ab^	<0.01 ^b^	<0.01 ^b^	0.01 ± 0.00 ^a^	<0.01 ^b^	<0.01 ^b^	<0.01 ^b^	<0.01 ^b^	<0.01 ^b^
[#28 *] Ethyl hexanoate isomer	2.26 ± 0.19 ^abc^	2.25 ± 0.03 ^abc^	2.38 ± 0.26 ^abc^	2.09 ± 0.42 ^bc^	2.36 ± 0.14 ^abc^	2.70 ± 0.41 ^abc^	2.34 ± 0.07 ^a^	1.74 ± 0.21 ^c^	3.11 ± 0.07 ^a^	2.24 ± 0.12 ^abc^	3.02 ± 0.41 ^ab^	2.53 ± 0.18 ^a^
[#29] Ethyl hexanoate isomer	0.18 ± 0.11 ^ab^	0.42 ± 0.21 ^ab^	0.24 ± 0.03 ^ab^	0.14 ± 0.14 ^ab^	0.39 ± 0.06 ^ab^	0.62 ± 0.17 ^a^	0.33 ± 0.05 ^a^	0.08 ± 0.07 ^b^	0.36 ± 0.13 ^ab^	0.49 ± 0.18 ^ab^	0.18 ± 0.14 ^ab^	0.28 ± 0.06 ^a^
[#33] Isoamyl butyrate	0.20 ± 0.02 ^a^	0.07 ± 0.01 ^cde^	0.09 ± 0.01 ^bcde^	0.11 ± 0.06 ^bcd^	0.14 ± 0.01 ^abc^	0.03 ± 0.01 ^e^	0.11 ± 0.01 ^a^	0.17 ± 0.02 ^ab^	0.04 ± 0.01 ^de^	0.12 ± 0.01 ^abc^	0.07 ± 0.02 ^cde^	0.10 ± 0.02 ^a^
[#35] Hexyl acetate	<0.01 ^a^	<0.01 ^a^	<0.01 ^a^	<0.01 ^a^	<0.01 ^a^	<0.01 ^a^	<0.01 ^a^	<0.01 ^a^	<0.01 ^a^	<0.01 ^a^	<0.01 ^a^	<0.01 ^a^
[#38] 2-Methylbutyl 2-methylbutyrate	0.09 ± 0.05	<0.01 ^c^	0.01 ± 0.00 ^c^	0.02 ± 0.02 ^bc^	0.19 ± 0.02 ^a^	<0.01 ^c^	0.05 ± 0.02 ^a^	0.06 ± 0.02 ^bc^	<0.01 ^c^	0.01 ± 0.01 ^c^	<0.01 ^c^	0.02 ± 0.01 ^a^
[#43] Propyl hexanoate	0.01 ± 0.01 ^ab^	0.02 ± 0.00 ^a^	<0.01 ^b^	<0.01 ^b^	0.02 ± 0.00 ^a^	<0.01 ^b^	0.01 ± 0.00 ^a^	<0.01 ^b^	<0.01 ^b^	0.01 ± 0.01 ^ab^	0.01 ± 0.00 ^ab^	<0.01 ^a^
[#45] Ethyl heptanoate	0.03 ± 0.03 ^ab^	0.03 ± 0.01 ^ab^	0.04 ± 0.02 ^ab^	<0.01 ^b^	0.03 ± 0.01 ^ab^	0.10 ± 0.06 ^a^	0.04 ± 0.01 ^a^	<0.01 ^b^	0.05 ± 0.01 ^ab^	0.02 ± 0.01 ^ab^	0.03 ± 0.03 ^ab^	0.02 ± 0.01 ^a^
[#50] Isobutyl hexanoate	0.01 ± 0.00 ^a^	<0.01 ^b^	<0.01 ^b^	<0.01 ^b^	<0.01 ^b^	<0.01 ^b^	<0.01 ^a^	<0.01 ^ab^	<0.01 ^b^	<0.01 ^b^	<0.01 ^b^	<0.01 ^a^
[#53] Allyl caproate	0.01 ± 0.00 ^a^	<0.01 ^b^	0.01 ± 0.00 ^a^	<0.01 ^b^	<0.01 ^b^	<0.01 ^b^	<0.01 ^a^	<0.01 ^b^	<0.01 ^b^	<0.01 ^b^	0.01 ± 0.01 ^a^	<0.01 ^a^
[#60] Butyl hexanoate	0.02 ± 0.00 ^a^	<0.01 ^c^	<0.01 ^bc^	<0.01 ^c^	0.01 ± 0.00 ^b^	<0.01 ^c^	0.01 ± 0.00 ^a^	<0.01 ^c^	0.01 ± 0.00 ^c^	<0.01 ^c^	0.01 ± 0.01 ^bc^	<0.01 ^a^
[#62 *] Ethyl caprylate	1.09 ± 0.17 ^ab^	1.48 ± 0.25 ^ab^	1.06 ± 0.31 ^ab^	0.52 ± 0.21 ^b^	1.13 ± 0.09 ^ab^	1.92 ± 0.99 ^a^	1.20 ± 0.14 ^a^	0.47 ± 0.12 ^b^	0.81 ± 0.06 ^ab^	0.70 ± 0.18 ^ab^	1.54 ± 0.09 ^ab^	0.88 ± 0.12 ^a^
[#65] Isoamyl caproate	0.10 ± 0.01 ^ab^	0.03 ± 0.01 ^cd^	0.03 ± 0.01 ^d^	0.03 ± 0.02 ^cd^	0.12 ± 0.01 ^a^	0.05 ± 0.02 ^bcd^	0.06 ± 0.01 ^a^	0.06 ± 0.02 ^bcd^	0.03 ± 0.01 ^cd^	0.09 ± 0.03 ^abc^	0.03 ± 0.01 ^d^	0.05 ± 0.01 ^a^
[#66] Methyl 2-hydroxy-4-methyl valerate	2.00 ± 0.05 ^a^	0.32 ± 0.02 ^d^	0.59 ± 0.06 ^c^	0.99 ± 0.14 ^b^	0.10 ± 0.04 ^ef^	0.27 ± 0.04 ^def^	0.71 ± 0.16 ^a^	0.31 ± 0.03 ^de^	0.31 ± 0.02 ^d^	0.08 ± 0.01 ^f^	0.32 ± 0.03 ^d^	0.26 ± 0.03 ^b^
[#69] Ethyl sorbate	<0.01 ^a^	<0.01 ^a^	<0.01 ^a^	0.01 ± 0.00 ^a^	<0.01 ^a^	<0.01 ^a^	<0.01 ^a^	<0.01 ^a^	<0.01 ^a^	<0.01 ^a^	<0.01 ^a^	<0.01 ^a^
[#73] Ethyl 2-hydroxy-4-methylpentanoate	0.02 ± 0.01 ^b^	<0.01 ^b^	0.01 ± 0.00 ^b^	0.02 ± 0.01 ^b^	0.01 ± 0.00 ^b^	0.01 ± 0.01 ^b^	0.01 ± 0.00 ^b^	0.01 ± 0.01 ^b^	0.06 ± 0.01 ^a^	0.01 ± 0.00 ^b^	0.02 ± 0.01 ^b^	0.03 ± 0.01 ^a^
[#77] Methyl caprate	0.01 ± 0.00 ^a^	<0.01 ^a^	<0.01 ^a^	<0.01 ^a^	<0.01 ^a^	<0.01 ^a^	<0.01 ^a^	<0.01 ^a^	<0.01 ^a^	<0.01 ^a^	<0.01 ^a^	<0.01 ^a^
[#82 *] Ethyl caprate	1.09 ± 0.20 ^a^	0.90 ± 0.10 ^abc^	0.41 ± 0.04 ^d^	0.36 ± 0.15 ^d^	1.33 ± 0.10 ^a^	1.33 ± 0.22 ^a^	0.90 ± 0.10 ^a^	0.31 ± 0.09 ^d^	0.55 ± 0.04 ^bcd^	0.49 ± 0.10 ^cd^	0.98 ± 0.04 ^ab^	0.58 ± 0.08 ^b^
[#85] Isoamyl octanoate	0.01 ± 0.01 ^a^	<0.01 ^b^	<0.01 ^b^	<0.01 ^b^	<0.01 ^b^	<0.01 ^b^	<0.01 ^a^	0.01 ± 0.01 ^ab^	<0.01 ^b^	<0.01 ^b^	<0.01 ^b^	<0.01 ^a^
[#87] Ethyl 9-decenoate	0.07 ± 0.01 ^abc^	0.03 ± 0.01 ^de^	0.02 ± 0.01 ^e^	0.05 ± 0.02 ^bcde^	0.06 ± 0.01 ^bcd^	0.05 ± 0.02 ^bcde^	0.05 ± 0.00 ^a^	0.07 ± 0.01 ^ab^	0.03 ± 0.00 ^cde^	0.10 ± 0.02 ^a^	0.04 ± 0.00 ^bcde^	0.06 ± 0.01 ^a^
[#89] 2-Ethoxyethyl butyrate	0.07 ± 0.01 ^ab^	<0.01 ^d^	0.04 ± 0.00 ^bcd^	0.09 ± 0.03 ^a^	0.05 ± 0.01 ^abc^	0.02 ± 0.00 ^cd^	0.04 ± 0.01 ^a^	0.06 ± 0.02 ^abc^	0.03 ± 0.01 ^bcd^	0.09 ± 0.02 ^d^	0.02 ± 0.00 ^cd^	0.05 ± 0.01 ^a^
[#91] Propyl decanoate	0.02 ± 0.00 ^b^	0.02 ± 0.00 ^b^	0.09 ± 0.02 ^a^	<0.01 ^b^	0.01 ± 0.00 ^b^	0.01 ± 0.01 ^b^	0.02 ± 0.01 ^a^	<0.01 ^b^	<0.01 ^b^	<0.01 ^b^	0.01 ± 0.01 ^b^	<0.01 ^b^
[#92] Benzyl acetate	<0.01 ^a^	<0.01 ^a^	<0.01 ^a^	<0.01 ^a^	<0.01 ^a^	<0.01 ^a^	<0.01 ^a^	<0.01 ^a^	<0.01 ^a^	<0.01 ^a^	<0.01 ^a^	<0.01 ^a^
[#94] Ethyl undecanoate	<0.01 ^a^	<0.01 ^a^	<0.01 ^a^	<0.01 ^a^	<0.01 ^a^	0.01 ± 0.00 ^a^	<0.01 ^a^	<0.01 ^a^	<0.01 ^a^	<0.01 ^a^	<0.01 ^a^	<0.01 ^a^
[#99] Ethyl phenylacetate	<0.01 ^a^	0.01 ± 0.00 ^a^	<0.01 ^a^	0.01 ± 0.01 ^a^	<0.01 ^a^	0.01 ± 0.01 ^a^	<0.01 ^a^	<0.01 ^a^	<0.01 ^a^	<0.01 ^a^	<0.01 ^a^	<0.01 ^a^
[#103] Phenethyl acetate	0.01 ± 0.00 ^cd^	<0.01 ^d^	0.01 ± 0.00 ^cd^	0.02 ± 0.01 ^bc^	0.01 ± 0.00 ^cd^	<0.01 ^d^	0.01 ± 0.00 ^b^	0.03 ± 0.00 ^a^	<0.01 ^d^	0.03 ± 0.01 ^ab^	0.01 ± 0.01 ^cd^	0.02 ± 0.00 ^a^
[#107] Isoamyl decanoate	0.01 ± 0.01 ^a^	<0.01 ^c^	<0.01 ^c^	<0.01 ^c^	0.01 ± 0.00 ^ab^	<0.01 ^c^	<0.01 ^a^	<0.01 ^bc^	<0.01 ^c^	<0.01 ^c^	<0.01 ^c^	<0.01 ^a^
[#110] Ethyl 3-phenylpropionate	<0.01 ^b^	0.02 ± 0.01 ^a^	<0.01 ^b^	<0.01 ^b^	<0.01 ^b^	<0.01 ^b^	<0.01 ^a^	<0.01 ^b^	<0.01 ^b^	0.01 ± 0.01 ^ab^	<0.01 ^b^	<0.01 ^a^
[#111] Dodecyl butyrate	0.10 ± 0.01 ^a^	0.01 ± 0.00 ^b^	<0.01 ^b^	<0.01 ^b^	0.01 ± 0.00 ^b^	<0.01 ^b^	0.02 ± 0.01 ^a^	<0.01 ^b^	0.02 ± 0.01 ^b^	0.01 ± 0.01 ^b^	0.01 ± 0.00 ^b^	0.01 ± 0.00 ^a^
[#127] Ethyl 4-octenoate	0.03 ± 0.01 ^a^	0.02 ± 0.01 ^a^	<0.01 ^b^	<0.01 ^b^	0.01 ± 0.00 ^ab^	0.02 ± 0.01 ^a^	0.01 ± 0.00 ^a^	0.01 ± 0.00 ^ab^	<0.01 ^b^	<0.01 ^b^	<0.01 ^b^	<0.01 ^b^
**Ester**	**16.27 ± 0.17 ^a^**	**10.78 ± 0.67 ^bcd^**	**9.18 ± 0.77 ^cd^**	**8.94 ± 0.64 ^d^**	**15.70 ± 0.86 ^a^**	**13.70 ± 1.67 ^ab^**	**12.43 ± 0.74 ^a^**	**8.85 ± 0.70 ^d^**	**12.12 ± 0.37 ^bc^**	**10.22 ± 0.52 ^cd^**	**11.26 ± 0.72 ^bcd^**	**10.61 ± 0.40 ^a^**
[#17] 1-Methoxy-2-propanol	0.10 ± 0.02 ^cde^	0.14 ± 0.01 ^bc^	0.11 ± 0.02 ^cde^	0.07 ± 0.03 ^cde^	0.12 ± 0.02 ^cd^	0.30 ± 0.03 ^a^	0.14 ± 0.02 ^a^	0.02 ± 0.01 ^e^	0.11 ± 0.02 ^cd^	0.03 ± 0.03 ^de^	0.23 ± 0.03 ^ab^	0.10 ± 0.03 ^a^
[#56] 3-Ethoxy-1-propanol	<0.01 ^b^	<0.01 ^b^	<0.01 ^b^	0.03 ± 0.01 ^a^	<0.01 ^b^	<0.01 ^b^	0.01 ± 0.00 ^a^	0.04 ± 0.02 ^a^	<0.01 ^b^	0.04 ± 0.00 ^a^	0.02 ± 0.01 ^ab^	0.02 ± 0.01 ^a^
[#101] 2-(2-Butoxyethoxy)Ethanol	<0.01 ^a^	<0.01 ^a^	<0.01 ^a^	<0.01 ^a^	<0.01 ^a^	<0.01 ^a^	<0.01 ^a^	<0.01 ^a^	<0.01 ^a^	<0.01 ^a^	<0.01 ^a^	<0.01 ^a^
[#79] γ -Valerolactone	<0.01 ^a^	<0.01 ^a^	<0.01 ^a^	<0.01 ^a^	<0.01 ^a^	<0.01 ^a^	<0.01 ^a^	<0.01 ^a^	<0.01 ^a^	<0.01 ^a^	<0.01 ^a^	<0.01 ^a^
[#88] γ-Caprolactone	<0.01 ^bc^	0.02 ± 0.01 ^a^	0.01 ± 0.00 ^abc^	<0.01 ^c^	<0.01 ^c^	<0.01 ^c^	0.01 ± 0.01 ^a^	<0.01 ^c^	0.02 ± 0.01 ^ab^	<0.01 ^c^	0.01 ± 0.00 ^abc^	0.01 ± 0.01 ^a^
[#100] δ-Caprolactone	0.02 ± 0.01 ^de^	0.10 ± 0.01 ^b^	0.13 ± 0.01 ^a^	0.03 ± 0.01 ^cd^	0.02 ± 0.00 ^de^	0.10 ± 0.01 ^b^	0.07 ± 0.05 ^a^	0.06 ± 0.01 ^c^	0.03 ± 0.00 ^d^	<0.01 ^e^	0.11 ± 0.01 ^ab^	0.05 ± 0.04 ^a^
[#119] δ-Octalactone	0.01 ± 0.00 ^c^	0.01 ± 0.00 ^c^	0.03 ± 0.01 ^ab^	0.02 ± 0.00 ^b^	0.01 ± 0.00 ^c^	0.01 ± 0.00 ^c^	0.01 ± 0.01 ^a^	0.01 ± 0.00 ^c^	0.03 ± 0.00 ^a^	0.01 ± 0.00 ^c^	0.02 ± 0.00 ^b^	0.02 ± 0.01 ^a^
[#122] 3,4,5-Trime thyldihydrofuran-2-one	0.02 ± 0.01 ^bc^	0.02 ± 0.01 ^cd^	0.02 ± 0.00 ^bcd^	0.01 ± 0.00 ^cd^	0.01 ± 0.00 ^cd^	0.03 ± 0.01 ^ab^	0.02 ± 0.01 ^a^	0.01 ± 0.01 ^d^	0.04 ± 0.00 ^a^	0.01 ± 0.00 ^cd^	0.02 ± 0.00 ^bcd^	0.02 ± 0.01 ^a^
[#123] γ-Decalactone	<0.01 ^b^	<0.01 ^b^	<0.01 ^b^	<0.01 ^b^	0.01 ± 0.00 ^a^	<0.01 ^b^	<0.01 ^a^	<0.01 ^b^	0.01 ± 0.01 ^a^	<0.01 ^b^	0.01 ± 0.00 ^a^	<0.01 ^a^
[#132] δ-Decalactone	0.04 ± 0.01 ^cde^	0.04 ± 0.01 ^bcde^	0.07 ± 0.01 ^a^	0.05 ± 0.00 ^abcd^	0.02 ± 0.01 ^e^	0.03 ± 0.01 ^de^	0.04 ± 0.01 ^a^	0.03 ± 0.01 ^e^	0.06 ± 0.01 ^abc^	0.04 ± 0.00 ^bcde^	0.06 ± 0.01 ^ab^	0.05 ± 0.02 ^a^
[#133] 3,4,5-Trimethyldihydrofuran-2-one	<0.01 ^ab^	<0.01 ^b^	<0.01 ^b^	<0.01 ^b^	<0.01 ^b^	<0.01 ^b^	<0.01 ^a^	<0.01 ^b^	0.01 ± 0.00 ^a^	<0.01 ^b^	<0.01 ^b^	<0.01 ^a^
[#137] γ-Dodecalactone	0.07 ± 0.01 ^c^	0.03 ± 0.00 ^ef^	0.03 ± 0.00 ^ef^	0.03 ± 0.01 ^f^	0.63 ± 0.02 ^a^	0.32 ± 0.01 ^b^	0.19 ± 0.23 ^a^	0.06 ± 0.01 ^cdef^	0.03 ± 0.01 ^def^	0.06 ± 0.00 ^cde^	0.06 ± 0.01 ^cd^	0.05 ± 0.01 ^a^
[#138] δ-Dodecalactone	0.01 ± 0.01 ^ab^	0.02 ± 0.01 ^ab^	0.02 ± 0.01 ^ab^	<0.01 ^b^	0.01 ± 0.01 ^ab^	0.02 ± 0.00 ^ab^	0.01 ± 0.01 ^a^	0.01 ± 0.00 ^ab^	0.01 ± 0.00 ^ab^	0.01 ± 0.00 ^ab^	0.03 ± 0.00 ^a^	0.02 ± 0.01 ^a^
**Furans**	**0.18 ± 0.02 ^de^**	**0.23 ± 0.04 ^d^**	**0.30 ± 0.01 ^c^**	**0.14 ± 0.00 ^e^**	**0.72 ± 0.02 ^a^**	**0.53 ± 0.01 ^b^**	**0.35 ± 0.05 ^a^**	**0.17 ± 0.02 ^e^**	**0.23 ± 0.02 ^d^**	**0.13 ± 0.00 ^e^**	**0.32 ± 0.01 ^c^**	**0.21 ± 0.02 ^b^**
[#2 *] 2-Butanone	12.89 ± 0.97 ^bc^	7.83 ± 0.27 ^d^	10.89 ± 0.27 ^c^	11.12 ± 0.93 ^c^	15.35 ± 0.26 ^b^	11.72 ± 0.82 ^c^	11.63 ± 2.39 ^b^	12.85 ± 0.19 ^c^	18.15 ± 0.93 ^a^	12.73 ± 0.67 ^c^	10.91 ± 0.44 ^c^	13.66 ± 2.87 ^a^
[#4] Diacetyl	0.08 ± 0.04 ^ab^	0.10 ± 0.02 ^ab^	0.11 ± 0.01 ^ab^	0.17 ± 0.05 ^a^	<0.01 ^b^	0.10 ± 0.01 ^ab^	0.09 ± 0.06 ^b^	0.17 ± 0.02 ^a^	0.17 ± 0.01 ^a^	0.11 ± 0.00 ^ab^	0.20 ± 0.08 ^a^	0.16 ± 0.05 ^a^
[#19] Acetyl valeryl	0.04 ± 0.00 ^a^	<0.01 ^a^	<0.01 ^a^	<0.01 ^a^	<0.01 ^a^	<0.01 ^a^	0.01 ± 0.02 ^a^	<0.01 ^a^	<0.01 ^a^	<0.01 ^a^	<0.01 ^a^	<0.01 ^a^
[#22] Butyl acetone	0.82 ± 0.08 ^ab^	0.33 ± 0.02 ^bc^	0.33 ± 0.01 ^bc^	0.24 ± 0.06 ^bc^	0.15 ± 0.02 ^c^	0.33 ± 0.11 ^bc^	0.37 ± 0.22 ^b^	0.54 ± 0.09 ^bc^	1.42 ± 0.47 ^a^	0.34 ± 0.10 ^bc^	0.36 ± 0.03 ^bc^	0.66 ± 0.51 ^a^
[#37 *] Acetoin	1.51 ± 0.05 ^d^	1.33 ± 0.10 ^d^	3.24 ± 0.21 ^a^	2.71 ± 0.28 ^ab^	1.74 ± 0.33 ^cd^	2.24 ± 0.09 ^bc^	2.13 ± 0.72 ^b^	2.78 ± 0.15 ^ab^	3.20 ± 0.21 ^a^	2.26 ± 0.11 ^bc^	2.24 ± 0.17 ^bc^	2.62 ± 0.44 ^a^
[#40] Acetol	0.01 ± 0.01 ^b^	0.01 ± 0.01 ^b^	0.04 ± 0.02 ^a^	0.02 ± 0.01 ^ab^	0.01 ± 0.00 ^b^	0.02 ± 0.00 ^ab^	0.02 ± 0.01 ^a^	0.03 ± 0.01 ^ab^	0.01 ± 0.00 ^b^	0.01 ± 0.01 ^ab^	0.02 ± 0.01 ^ab^	0.02 ± 0.01 ^a^
[#46] Methylheptenone	0.02 ± 0.01 ^ab^	0.01 ± 0.01 ^ab^	0.02 ± 0.01 ^ab^	0.03 ± 0.00 ^a^	<0.01 ^b^	<0.01 ^b^	0.01 ± 0.01 ^a^	0.01 ± 0.00 ^ab^	0.02 ± 0.01 ^ab^	<0.01 ^b^	0.03 ± 0.01 ^a^	0.02 ± 0.01 ^a^
[#47] 3-Hydroxy-2-pentanone	0.16 ± 0.03 ^c^	0.24 ± 0.06 ^bc^	0.47 ± 0.09 ^a^	0.26 ± 0.02 ^bc^	0.20 ± 0.04 ^c^	0.21 ± 0.01 ^c^	0.26 ± 0.11 ^a^	0.31 ± 0.03 ^bc^	0.38 ± 0.03 ^ab^	0.25 ± 0.02 ^bc^	0.26 ± 0.02 ^bc^	0.30 ± 0.06 ^a^
[#51] 2-Hydroxy-3-pentanone	0.15 ± 0.02 ^c^	0.23 ± 0.06 ^abc^	0.34 ± 0.03 ^a^	0.32 ± 0.05 ^ab^	0.20 ± 0.03 ^bc^	0.22 ± 0.01 ^abc^	0.24 ± 0.07 ^a^	0.30 ± 0.03 ^ab^	0.22 ± 0.03 ^abc^	0.25 ± 0.03 ^abc^	0.23 ± 0.02 ^abc^	0.25 ± 0.04 ^a^
[#57 *] 2-Nonanone	1.53 ± 0.53 ^a^	0.25 ± 0.02 ^b^	0.22 ± 0.08 ^b^	0.15 ± 0.09 ^b^	0.13 ± 0.06 ^b^	0.27 ± 0.22 ^b^	0.42 ± 0.55 ^a^	0.42 ± 0.21 ^ab^	1.52 ± 0.68 ^a^	0.42 ± 0.29 ^ab^	0.16 ± 0.06 ^b^	0.63 ± 0.64 ^a^
[#63] 5-Nonen-2-one	<0.01 ^b^	0.01 ± 0.01 ^a^	<0.01 ^b^	<0.01 ^b^	<0.01 ^b^	<0.01 ^b^	<0.01 ^a^	<0.01 ^b^	<0.01 ^b^	<0.01 ^b^	<0.01 ^b^	<0.01 ^a^
[#67] 2-Decanone	0.01 ± 0.00 ^a^	<0.01 ^b^	<0.01 ^b^	<0.01 ^b^	<0.01 ^b^	<0.01 ^b^	<0.01 ^a^	<0.01 ^b^	0.01 ± 0.01 ^a^	<0.01 ^b^	<0.01 ^b^	<0.01 ^a^
[#78] 2-Undecanone	0.16 ± 0.06 ^a^	0.01 ± 0.01 ^b^	0.01 ± 0.00 ^b^	0.01 ± 0.01 ^b^	0.01 ± 0.00 ^b^	0.02 ± 0.02 ^b^	0.04 ± 0.06 ^a^	0.01 ± 0.01 ^b^	0.06 ± 0.02 ^b^	0.02 ± 0.01 ^b^	0.01 ± 0.00 ^b^	0.02 ± 0.02 ^a^
[#83] Acetophenone	0.01 ± 0.01 ^b^	0.03 ± 0.01 ^a^	0.02 ± 0.01 ^ab^	0.01 ± 0.00 ^ab^	<0.01 ^b^	0.01 ± 0.01 ^ab^	0.01 ± 0.01 ^a^	<0.01 ^b^	0.01 ± 0.00 ^ab^	<0.01 ^b^	0.02 ± 0.00 ^ab^	0.01 ± 0.01 ^a^
[#106] Nerylacetone	0.01 ± 0.00 ^a^	0.01 ± 0.01 ^b^	<0.011 ^b^	0.01 ± 0.01 ^b^	<0.01 ^b^	<0.01 ^b^	0.01 ± 0.01 ^a^	<0.01 ^b^	<0.01 ^b^	<0.01 ^b^	<0.01 ^b^	<0.01 ^b^
**Ketone**	**17.39 ± 0.62 ^bc^**	**10.39 ± 0.25 ^f^**	**15.69 ± 0.02 ^cde^**	**15.04 ± 0.66 ^de^**	**17.78 ± 0.11 ^b^**	**15.15 ± 0.46 ^de^**	**15.24 ± 0.59 ^b^**	**17.42 ± 0.25 ^b^**	**25.17 ± 0.83 ^a^**	**16.39 ± 0.22 ^bcd^**	**14.44 ± 0.32 ^e^**	**18.35 ± 1.24 ^a^**
[#7] α-Pinene	0.02 ± 0.01 ^b^	<0.01 ^c^	0.03 ± 0.01 ^a^	<0.01 ^c^	<0.01 ^c^	<0.01 ^c^	0.01 ± 0.01 ^a^	<0.01 ^c^	<0.01 ^c^	<0.01 ^c^	<0.01 ^c^	<0.01 ^b^
[#23] D-Limonene	0.35 ± 0.06 ^a^	0.19 ± 0.03 ^a^	0.29 ± 0.12 ^a^	0.19 ± 0.09 ^a^	0.14 ± 0.03 ^a^	0.16 ± 0.09 ^a^	0.22 ± 0.10 ^a^	0.14 ± 0.09 ^a^	0.21 ± 0.03 ^a^	0.07 ± 0.04 ^a^	0.20 ± 0.14 ^a^	0.16 ± 0.09 ^a^
[#24] Eucalyptol	0.02 ± 0.02 ^a^	0.02 ± 0.01 ^a^	0.04 ± 0.01 ^a^	0.01 ± 0.01 ^a^	<0.01 ^a^	0.03 ± 0.02 ^a^	0.02 ± 0.02 ^a^	0.01 ± 0.01 ^a^	0.01 ± 0.01 ^a^	0.01 ± 0.01 ^a^	0.01 ± 0.01 ^a^	0.01 ± 0.01 ^b^
**Terpene**	**0.38 ± 0.08 ^a^**	**0.22 ± 0.02 ^a^**	**0.35 ± 0.13 ^a^**	**0.20 ± 0.08 ^a^**	**0.14 ± 0.03 ^a^**	**0.19 ± 0.10 ^a^**	**0.25 ± 0.03 ^a^**	**0.15 ± 0.10 ^a^**	**0.22 ± 0.03 ^a^**	**0.08 ± 0.05 ^a^**	**0.21 ± 0.14 ^a^**	**0.16 ± 0.03 ^b^**
[#55] Dimethyltrisulfide	<0.01 ^b^	0.04 ± 0.02 ^a^	0.02 ± 0.01 ^ab^	<0.01 ^b^	<0.01 ^b^	<0.01 ^b^	0.01 ± 0.02 ^a^	<0.01 ^b^	<0.01 ^b^	<0.01 ^b^	<0.01 ^b^	<0.01 ^a^
[#71] Dihydro-2-methyl-3(2H)-thiophenone	<0.01 ^c^	<0.01 ^c^	<0.01 ^c^	0.02 ± 0.01 ^b^	<0.01 ^c^	<0.01 ^c^	<0.01 ^a^	<0.01 ^c^	<0.01 ^c^	0.03 ± 0.01 ^a^	<0.01 ^c^	0.01 ± 0.01 ^a^
[#112] Dymethyl sulfone	0.06 ± 0.01 ^abc^	0.04 ± 0.00 ^cd^	0.06 ± 0.01 ^abc^	0.05 ± 0.01 ^bc^	0.05 ± 0.01 ^bc^	0.05 ± 0.01 ^bc^	0.05 ± 0.01 ^a^	0.07 ± 0.01 ^ab^	0.02 ± 0.00 ^d^	0.08 ± 0.01 ^a^	0.04 ± 0.00 ^cd^	0.05 ± 0.02 ^a^
[#117] Phenylacetonitrile	0.01 ± 0.00 ^ab^	<0.01 ^b^	<0.01 ^b^	<0.01 ^b^	<0.01 ^b^	<0.01 ^b^	<0.01 ^b^	0.02 ± 0.01 ^a^	<0.01 ^b^	0.02 ± 0.01 ^ab^	<0.01 ^b^	0.01 ± 0.01 ^a^
**Minor**	**0.07 ± 0.01 ^bcd^**	**0.08 ± 0.02 ^bc^**	**0.08 ± 0.01 ^bc^**	**0.06 ± 0.01 ^bcd^**	**0.05 ± 0.01 ^cde^**	**0.05 ± 0.01 ^cde^**	**0.07 ± 0.00 ^a^**	**0.09 ± 0.00 ^ab^**	**0.02 ± 0.00 ^e^**	**0.12 ± 0.02 ^a^**	**0.04 ± 0.00 ^de^**	**0.07 ± 0.01 ^a^**

## Data Availability

The original contributions presented in this study are included in the article and Supplementary Materials. Further inquiries can be directed to the corresponding author.

## Supplementary Materials

The following supporting information can be downloaded at: https://www.mdpi.com/article/10.3390/foods14091509/s1. Table S1: Retention time of the compounds in the alkane mixture, C8–C20, analyzed by GC-MS; Table S2: Volatile compounds tentatively identified in Serpa cheese samples, their corresponding linear retention indexes (LRI), odor descriptors, odor threshold, and similarity index (SI); Table S3: Pearson’s correlation coefficients between the relative volatile compound area and the sensory evaluation made by the sensory panel for the Serpa cheese samples; Table S4: Corresponding loading values in factor 1 and factor 2, obtained by PLS-DA.
