# Supplementary material for: Matching the Sensory Analysis of Serpa PDO Cheese with the Volatile Profiles—A Preliminary Study
Source: Foods. 2025 Apr 25;14(9):1509. doi: 10.3390/foods14091509 (PMC12072146; doi:10.3390/foods14091509)
Supplement: Supplementary file 1 [file foods-14-01509-s001.zip › Supplementary file/Tables S1.docx]

**Supplementary material**

**Table S1.** Retention time of the compounds in the alkane mixture, C8 – C20, analysed by GCMS

| Alkane | Retention time (min) |
| --- | --- |
| C8 | 2.990 |
| C9 | 4.249 |
| C10 | 6.808 |
| C11 | 10.121 |
| C12 | 13.460 |
| C13 | 16.591 |
| C14 | 19.500 |
| C15 | 22.217 |
| C16 | 24.777 |
| C17 | 27.196 |
| C18 | 29.500 |
| C19 | 31.609 |
| C20 | 32.848 |
