# Supplementary material for: Matching the Sensory Analysis of Serpa PDO Cheese with the Volatile Profiles—A Preliminary Study
Source: Foods. 2025 Apr 25;14(9):1509. doi: 10.3390/foods14091509 (PMC12072146; doi:10.3390/foods14091509)
Supplement: Supplementary file 1 [file foods-14-01509-s001.zip › Supplementary file/Tables S4.docx]

**Supplementary material**

**Table S4.** Correlation loading values in factor 1 and factor 2, obtained by PLS-DA

| **Volatile Compound #** | **Factor-1** | **Factor-2** |
| --- | --- | --- |
| 1 | 0.0142 | -0.2982 |
| 2 | -0.3903 | -0.3130 |
| 3 | -0.2131 | -0.1462 |
| 4 | -0.5649 | -0.1440 |
| 5 | 0.4891 | -0.0774 |
| 6 | 0.5651 | -0.3149 |
| 7 | 0.5085 | -0.1474 |
| 8 | 0.4013 | -0.2354 |
| 9 | 0.3668 | -0.2588 |
| 10 | 0.6765 | -0.0709 |
| 11 | 0.0045 | 0.2052 |
| 12 | -0.2586 | -0.2058 |
| 13 | 0.8574 | -0.0027 |
| 14 | 0.0771 | 0.1742 |
| 15 | -0.2604 | 0.0112 |
| 16 | 0.4271 | -0.4133 |
| 17 | 0.3992 | -0.1433 |
| 18 | -0.2841 | 0.4829 |
| 19 | 0.5279 | -0.4228 |
| 20 | 0.0781 | 0.0288 |
| 21 | -0.5998 | -0.0269 |
| 22 | -0.1879 | -0.7133 |
| 23 | 0.6642 | -0.3268 |
| 24 | 0.5770 | -0.0953 |
| 25 | 0.5279 | -0.4228 |
| 26 | -0.2939 | 0.4867 |
| 27 | 0.3109 | 0.2476 |
| 28 | -0.0397 | -0.4926 |
| 29 | 0.5508 | -0.3781 |
| 30 | 0.5313 | -0.4221 |
| 31 | -0.3500 | 0.4659 |
| 32 | 0.5803 | -0.2722 |
| 33 | 0.0839 | 0.0222 |
| 34 | -0.0562 | -0.1143 |
| 35 | -0.4883 | -0.0065 |
| 36 | 0.5092 | -0.5559 |
| 37 | -0.5564 | 0.0860 |
| 38 | 0.2512 | 0.1572 |
| 39 | -0.2859 | -0.1583 |

**Table S4:** *Cont*.

| **Volatile Compound #** | **Factor-1** | **Factor-2** |
| --- | --- | --- |
| 40 | -0.1482 | 0.2690 |
| 41 | 0.5755 | -0.2429 |
| 42 | 0.3609 | 0.0013 |
| 43 | 0.4118 | -0.0246 |
| 44 | -0.0239 | -0.5125 |
| 45 | 0.3786 | -0.2888 |
| 46 | -0.0419 | 0.0075 |
| 47 | -0.3012 | -0.0457 |
| 48 | -0.0033 | -0.0808 |
| 49 | 0.5963 | -0.1991 |
| 50 | 0.2702 | 0.0667 |
| 51 | -0.6190 | 0.5873 |
| 52 | 0.4279 | -0.0605 |
| 53 | 0.4584 | -0.3703 |
| 54 | 0.2171 | 0.1104 |
| 55 | 0.4709 | 0.0279 |
| 56 | -0.7471 | 0.3475 |
| 57 | 0.0549 | -0.7106 |
| 58 | 0.2494 | -0.0408 |
| 59 | 0.0008 | 0.1165 |
| 60 | 0.3776 | -0.5728 |
| 61 | 0.3462 | -0.1261 |
| 62 | 0.5646 | -0.2107 |
| 63 | 0.3785 | -0.0269 |
| 64 | -0.7403 | -0.0602 |
| 65 | 0.1418 | -0.0320 |
| 66 | 0.5275 | -0.1363 |
| 67 | 0.2515 | -0.6522 |
| 68 | -0.0963 | 0.1200 |
| 69 | -0.0993 | 0.7315 |
| 70 | 0.5906 | -0.6806 |
| 71 | -0.4921 | 0.4022 |
| 72 | 0.4899 | 0.1597 |
| 73 | -0.3887 | -0.4534 |
| 74 | -0.3021 | 0.1410 |
| 75 | -0.6573 | -0.0398 |
| 76 | -0.3295 | 0.3012 |
| 77 | 0.5279 | -0.4228 |
| 78 | 0.4161 | -0.5565 |
| 79 | 0.0406 | -0.3122 |

**Table S4:** *Cont*.

| **Volatile Compound #** | **Factor-1** | **Factor-2** |
| --- | --- | --- |
| 80 | -0.6957 | 0.2929 |
| 81 | 0.2198 | 0.0634 |
| 82 | 0.5892 | -0.1699 |
| 83 | 0.3940 | -0.1242 |
| 84 | -0.2361 | 0.5249 |
| 85 | 0.3211 | -0.4006 |
| 86 | -0.5011 | 0.2516 |
| 87 | -0.3715 | 0.0552 |
| 88 | 0.2057 | -0.4860 |
| 89 | -0.3635 | 0.3666 |
| 90 | -0.6737 | 0.2858 |
| 91 | 0.4871 | 0.0137 |
| 92 | 0.5279 | -0.4228 |
| 93 | 0.3150 | -0.0542 |
| 94 | 0.2171 | 0.1104 |
| 95 | 0.5755 | -0.2429 |
| 96 | -0.0128 | -0.3968 |
| 97 | 0.3586 | -0.0587 |
| 98 | -0.2679 | 0.0561 |
| 99 | 0.4532 | 0.4890 |
| 100 | 0.3174 | 0.0021 |
| 101 | -0.0993 | 0.7315 |
| 102 | 0.1818 | -0.0059 |
| 103 | -0.5719 | 0.2762 |
| 104 | 0.1138 | -0.1336 |
| 105 | -0.0332 | 0.0716 |
| 106 | 0.6695 | 0.0621 |
| 107 | 0.4470 | -0.1894 |
| 108 | 0.4997 | 0.0030 |
| 109 | 0.1209 | 0.1208 |
| 110 | 0.2195 | -0.0035 |
| 111 | 0.4624 | -0.5328 |
| 112 | -0.0979 | 0.1582 |
| 113 | -0.1360 | 0.0252 |
| 114 | 0.1522 | 0.2713 |
| 115 | 0.0777 | 0.6306 |
| 116 | 0.3484 | 0.0104 |
| 117 | -0.4559 | -0.1957 |
| 118 | 0.3554 | 0.0025 |
| 119 | -0.2268 | -0.1317 |

**Table S4:** *Cont*.

| **Volatile Compound #** | **Factor-1** | **Factor-2** |
| --- | --- | --- |
| 120 | 0.1895 | 0.2793 |
| 121 | 0.0375 | -0.1742 |
| 122 | 0.1803 | -0.5596 |
| 123 | -0.1911 | -0.2405 |
| 124 | 0.2180 | 0.4096 |
| 125 | 0.4856 | -0.2147 |
| 126 | 0.3616 | 0.1640 |
| 127 | 0.7313 | -0.2122 |
| 128 | -0.1098 | 0.7570 |
| 129 | -0.0956 | 0.7274 |
| 130 | 0.1609 | 0.0480 |
| 131 | -0.2011 | 0.2134 |
| 132 | -0.0659 | -0.1612 |
| 133 | -0.1289 | -0.6189 |
| 134 | 0.4545 | 0.3096 |
| 135 | 0.5587 | -0.0270 |
| 136 | 0.5753 | 0.0255 |
| 137 | 0.2347 | 0.2638 |
| 138 | 0.2394 | -0.4749 |
| 139 | 0.4586 | -0.0203 |
| 140 | -0.1868 | 0.3822 |
| 141 | 0.6025 | -0.0395 |
| 142 | 0.5279 | -0.4228 |
| 143 | 0.1311 | -0.0872 |
| 144 | 0.2967 | 0.4347 |
| **High Score** | -0.8671 | -0.4669 |
| **Low Score** | 0.8671 | 0.4669 |
